# Fibrogenic fibroblast-selective near-infrared phototherapy to control scarring

**DOI:** 10.7150/thno.36375

**Published:** 2019-09-19

**Authors:** Zelin Chen, Ziwen Wang, Taotao Jin, Gufang Shen, Yu Wang, Xu Tan, Yibo Gan, Fan Yang, Yunsheng Liu, Chunji Huang, Yixin Zhang, Xiaobing Fu, Chunmeng Shi

**Affiliations:** 1 Institute of Rocket Force Medicine, State Key Laboratory of Trauma, Burns and Combined Injury, Army Medical University, Chongqing, 400038, China; 2 College of Basic Medical Sciences, Army Medical University, 400038 Chongqing, China.; 3 Department of Plastic and Reconstructive Surgery Shanghai Ninth People's Hospital Shanghai Jiao Tong University, School of Medicine, Shanghai, 200011, China; 4 Institute of Basic Medical Sciences, Chinese PLA General Hospital, Beijing, 100853, China

**Keywords:** fibroblasts, scarring, phototherapy

## Abstract

**Rationale**: Fibroblasts, the predominant cell type responsible for tissue fibrosis, are heterogeneous, and the targeting of unique fibrogenic population of fibroblasts is highly expected. Very recently, elevated glycolysis is demonstrated to play a pivotal role in the determination of fibrogenic phenotype of fibroblasts. However, it is lack of specific strategies for targeting and elimination of such fibrogenic populations. In this study, a novel strategy to use the a near-infrared (NIR) dye IR-780 for the targeting and elimination of a fibrogenic population of glycolytic fibroblasts to control the cutaneous scarring is developed.

**Methods:** The identification and cell properties test of fibrogenic fibroblasts with IR-780 were conducted by using fluorescence activated cell sorting, transplantation experiments, *in vivo* imaging, RNA sequencing in human cell experiments and mouse and rat wound models. The uptake of IR-780 in fibroblasts mediated by HIF-1α/SLCO2A1 and the metabolic properties of IR-780^H^ fibroblasts were investigated using RNA interference or signaling inhibitors. The fibrogenic fibroblast-selective near-infrared phototherapy of IR-780 were evaluated in human cell experiments and mouse wound models.

**Results:** IR-780 is demonstrated to recognize a unique glycolytic fibroblast lineage, which is responsible for the bulk of connective tissue deposition during cutaneous wound healing and cancer stroma formation. Further results identified that SLCO2A1 is involved in the preferential uptake of IR-780 in fibrogenic fibroblasts, which is regulated by HIF-1α. Moreover, with intrinsic dual phototherapeutic activities, IR-780 significantly diminishes cutaneous scarring through the targeted ablation of the fibrogenic population by photothermal and photodynamic effects.

**Conclusion:** This work provides a unique strategy for the targeted control of tissue scarring by fibrogenic fibroblast-selective near-infrared phototherapy. It is proposed that IR-780 based theranostic methodology holds promise for translational medicine aimed at regulation of fibrogenic behavior.

## Introduction

Tissue fibrosis is characteristic features of many human diseases and major causes of morbidity and mortality worldwide. Currently, treatment of tissue fibrosis is severely limited, and organ transplantation is often the only effective option for end-stage fibrotic diseases. However, limited donor organ availability and the high cost and morbidity of transplantation underscore the urgent need for more effective therapies^1^. Fibroblasts are the principal mesenchymal cell type in connective tissue that deposits the extracellular matrix (ECM) in both embryonic and adult organs^2^ , are the predominant cell type responsible for cutaneous scarring^3^, tissue and organ fibrosis^4^ and tumor stroma contribution^5^. Recently, there is increasing evidence that even in a single tissue, fibroblasts exhibit significant functional heterogeneity^6^, ^7^ . Several studies have preliminarily demonstrated that specific fibroblast subpopulation(s) are proposed to be responsible for the development and progression of fibrosis 8-12. However, so far, there still lack effective practical strategies for the targeting these cell populations. Recent reports showed that fibroblasts exhibited enhanced aerobic glycolysis in fibrosis related diseases such as skin fibrosis^13^, cancer^5^, laryngotracheal stenosis^14^, keloid^15^, pulmonary hypertension^16^, idiopathic pulmonary fibrosis^17^, et al. Whether enhanced glycolysis be one of the most important properties of fibrogenic fibroblast population is still unclear. In our previous work, we have identified a NIR small molecule dye IR-780, which could selectively accumulate in the glycolytic tumor cells^18^. Whether IR-780 could identify the diversity of glycolytic activity in normal and pathological cells and target the glycolytic fibroblast population in tissue fibrosis, and whether the glycolytic fibroblast population be fibrogenic potential are still largely unknown. Interestingly, in this study, IR-780 could selectively identify a hyperactive glycolysis fibroblast lineage and this population is the primary contributor to connective tissue secretion and organization during cutaneous wounding and cancer stroma formation, which demonstrated that hyperactive glycolysis would be an important feature of fibrogenic fibroblast population. Moreover, IR-780, as a photosensitizing agent, is shown to process photothermal (PTT) and photodynamic (PDT) properties. In addition, IR-780 could target the fibrogenic fibroblast population, and it is quietly different from most existing photosensitizers which are poorly selective small molecules. Further, IR-780 based NIR phototherapy selectively ablated the fibrogenic fibroblast population *in vitro,* and sharply reduced the numbers of myofibroblasts and ECM production. Moreover, the IR-780 based NIR phototherapy is shown none tested side effects, which promises this fibrogenic fibroblast-selective phototherapeutic strategy as a potential treatment of tissue fibrosis.

## Materials and Methods

### Animals and wound model

6-10 weeks old male and female SD rats were used for human fibroblasts transplanting wound models. 6-10 weeks old male and female C57/BL mice were used for cutaneous wound models and granulation tissue cell isolation. Newborn ROSA26^mTmG^ mice form the Jackson Laboratory were used for neonatal fibroblast isolation. Wound models were performed previously 19. Briefly, mice or rats were anesthetized with 1% pentobarbital (30 mg/kg). The back hair was shaved. Circular, full-thickness skin excisions of 10 mm in diameter were surgically made in the middle back of each animal. *in vivo* experiments were conducted in accordance with the Guidelines for the Care and Use of Laboratory Animals of the AMU, and all procedures were approved by the Animal Care and Use Committee of the AMU.

### Cell isolation and culture

Human foreskins were obtained after prepucectomy of foreskins and approval of the protocol by the ethics committee of Army Medical University. The granulation tissues were harvested at 7 days after wounding. The isolation protocols of human fibroblasts, granulation tissue cells and neonatal ROSA26^mTmG^ mouse fibroblasts are described previously^20^. In Brief, skin tissues of 1-2 cm^2^ pieces with subcutaneous tissue removal were digested over night at 4°C in a digestion medium containing 1mg/mL dispase (Roche). Following stripping the epidermis, the dermis were cut up and incubated in the digestion medium consisting of DMEM with 0.25% collagenase I (Worthington) at 37 ℃for 1 hour with shaking. The digested cells were then passed through a 75-μm cell strainer, centrifuged, and resuspended in DMEM with 10% foetal bovine serum (Hyclone), 100 U/mL penicillin, and 0.1 mg/mL streptomycin (Beyotime).

### Subcellular Localization of IR-780

2×10^5^ human or mouse fibroblasts were seeded in a 35 mm petri dish and cultured overnight. Cells were incubated with 1μM IR-780 in DMEM for 20 min at 37 °C, then stained with Mito-tracker (1:7000 diluted with PBS) for another 15 min at 37 °C, following stained by Hoechst 33342 for 10 min at room temperature (RT). Finally, fluorescence of cells was recorded by the Leica confocal microscope after being rinsed with PBS. The whole stained procedure was carried out in the dark condition.

### *In vitro* cell uptake analysis of IR-780

2×10^6^ human or mouse fibroblasts were seeded in 30 mm dishes and cultured overnight. To test the factors that would affect the uptake of IR-780, cells were treated with different factors: 1) aerobic glycolysis: Cells were treated with 2-Deoxy-D-glucose (2-DG, 150mM) for 45 min or 6-aminonicotinamide (6-AN,5μm,Sigma) for 24 hours or 3-(3-Pyridinyl)-1-(4-pyridinyl)-2-propen-1-one (3PO,10 μM, Sigma) for 24 hours respectively. 2) organic-anion-transporting polypeptides (OATPs): Cells were treated with sulfobromophthalein disodium salt hydrate (BSP, 250μM, Sigma) for 5min or SLCO2A1, SLCO1B3 siRNA for 48 hours. 3) HIF-1a pathway: Cells were treated with CTGF siRNA for 48 hours or 5% hypoxia for 48 hours or 5% hypoxia+LW6 (a HIF-1α inhibitor, 10μM, Selleck) for 48 hours. 4) endocytosis: Cells were treated with amiloride(50μg/mL) or chlorpromazine (3.75μg/mL) or methyl-β-cyclodextrin (4×10^-3^M ) for 1 hour respectively. Cells were then incubated with 1μM IR-780 in DMEM for 20 min at 37°C. Following thrice washes with PBS, NIR images were directly captured by fluorescent microscope (Leica, excitation/emission: 770/830). Or cells were fixed by 4% polyoxymethylene for 15 min at RT and stained with DAPI for 1 min. NIR images were then captured.

### Fluorescence activated cell sorting (FACS) of IR-780 labeled fibroblasts

Primary human/neonatal ROSA26^mTmG^ fibroblasts or mouse granulation cells were cultured to confluence and washed thrice with PBS. Cells were incubated in DMEM+IR-780 (1μM) at 37 ℃ for 20 min in CO_2_ incubator and then washed thrice with PBS. Labeled cells were collected in PBS at a density of about 2-3×10^7^/ml. After passing through a 75-μm cell strainer, cells were sorting by Beckman Moflo XDP flow cytometer with 650 nm excitation and 780 nm emission. For *in vivo* IR-780 labeled granulation tissue cells sorting, mice at 6^th^ day after wounding were received intraperitoneal injection of IR-780 (1.334 mg/kg) in 0.2 ml PBS. 24 hours later, granulation tissue cells were isolated and received directly FACS as described above. Following each sorting, a small part of IR-780^H^ and control fibroblasts were seeded in 30mm dishes and cultured for 12 hours and then fixed by 4% polyoxymethylene for 15 min at RT and stained with DAPI for 1 min. Cells were then rinsed with PBS for NIR images capture with the NIR fluorescent microscope.

### Colony-forming unit fibroblast assay

FACS-sorted IR-780^H^ or control human fibroblasts were seeded into six-well plates at a density of 1000 cells per well in six-well plates and cultured for 12 days with medium replaced every 3 days. Colonies were fixed by 4% polyoxymethylene for 15 min at RT and stained with crystal violet for 10 min. After thrice washes with PBS, colonies and counted as previously described^20^.

### TGF-β1 response

FACS-sorted IR-780^H^ and control human fibroblasts were seeded in the 24-well plates covered with slides (for immunofluorescence) and 60 mm dishes (for real- time PCR) and cultured overnight. Cells were then treated with *TGF-β1*(5 ng/ml) and harvested for immunofluorescence and real-time PCR at 6, 12, 24 hours after *TGF-β1* stimulation.

### * In vivo* detection of IR-780

Wounded mice were intraperitoneally injected with IR-780 (1.334mg/kg) at 2, 6,14 days following wounding respectively. 24 hours later, mice were anesthetized by 1% pentobarbital sodium (Sigma) and the whole body NIR fluorescent imaging was taken using a Kodak In-Vivo FX Profession Imaging System equipped with fluorescent filter sets (excitation/emission, 770/830 nm). Fluorescent images were co-registered with the anatomical X-ray images in this system. Following, wounded tissues at each time point were harvested respectively for NIR fluorescent imaging. Then cytosections of each wound tissue were co-stained with collagen and DAPI (described in supplemental information 'Immunofluorescence and immunohistochemistry' section). Fluorescent images were captured on a Leica confocal microscope.

### Statistical analysis

Statistical analyses were performed using the SPSS 13.0 package. Results were expressed as the means±S.D. An independent-samples t-test was used to determine significant differences between two groups. Comparisons of multiple groups were performed using one-way analysis of variance with corrections for multiple comparisons. P<0.05 was statistically significant.

## Results

### IR-780^H^ Fibroblast Population Exhibits High Fibrogenic Potential *in vitro*

**Figure 1:**
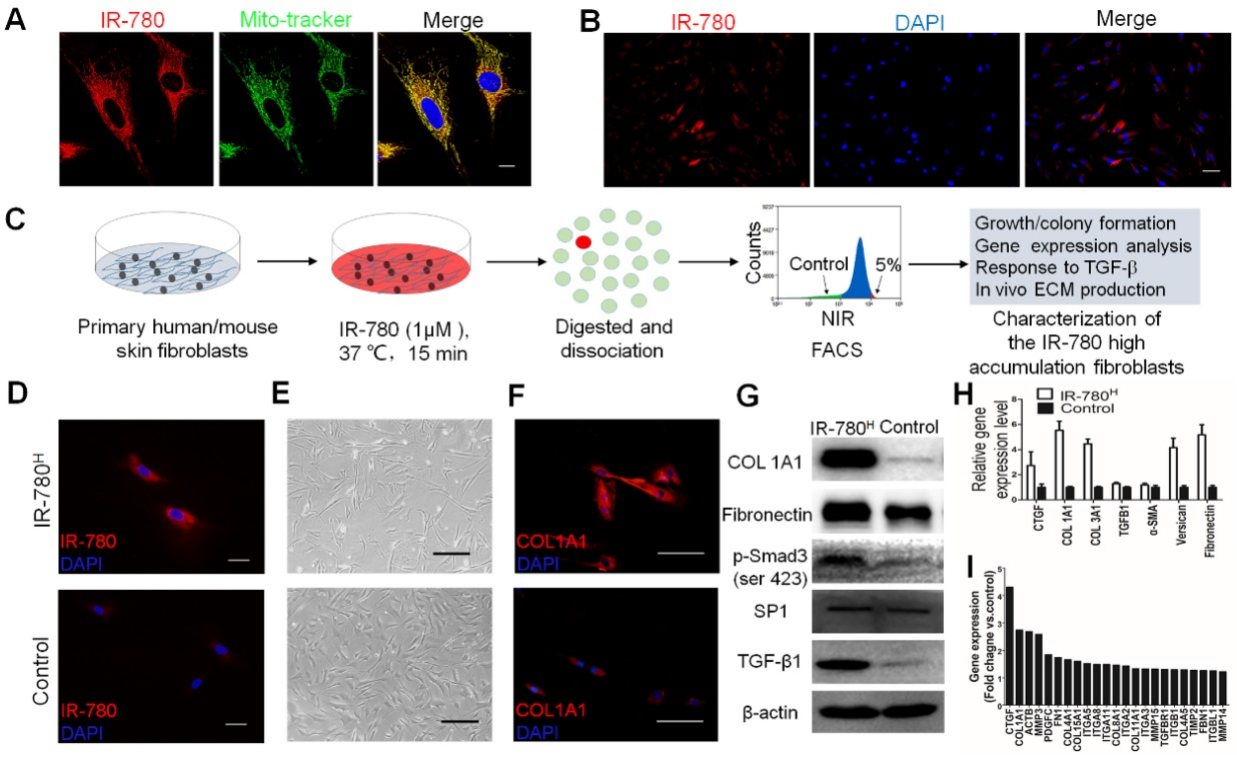
** Isolation and characterization of fibrogenic fibroblasts by IR-780.** (A) the subcellular localization of IR-780 in skin fibroblasts. Scale bar, 25 µm. (B) the differentially accumulation of R-780 in the human skin fibroblasts. Scale bar, 75 µm. (C) Schematic of the experimental strategy for isolation and characterization of IR-780^H^ fibroblasts. (D) the NIR images of newly isolated IR-780^H^ and control fibroblasts. Scale bar, 75 µm. (E) the morphologic images of IR-780^H^ and control fibroblasts. Scale bar, 100 µm.(F) Immunostaining of IR-780^H^ and control fibroblasts for *COL1A1*. Scale bar, 100 µm.(G) western blots, (H) real-time RT-PCR and (I) gene expression profile analyzing the fibrogenic genes in IR-780^H^ and control fibroblasts.

**Figure 2:**
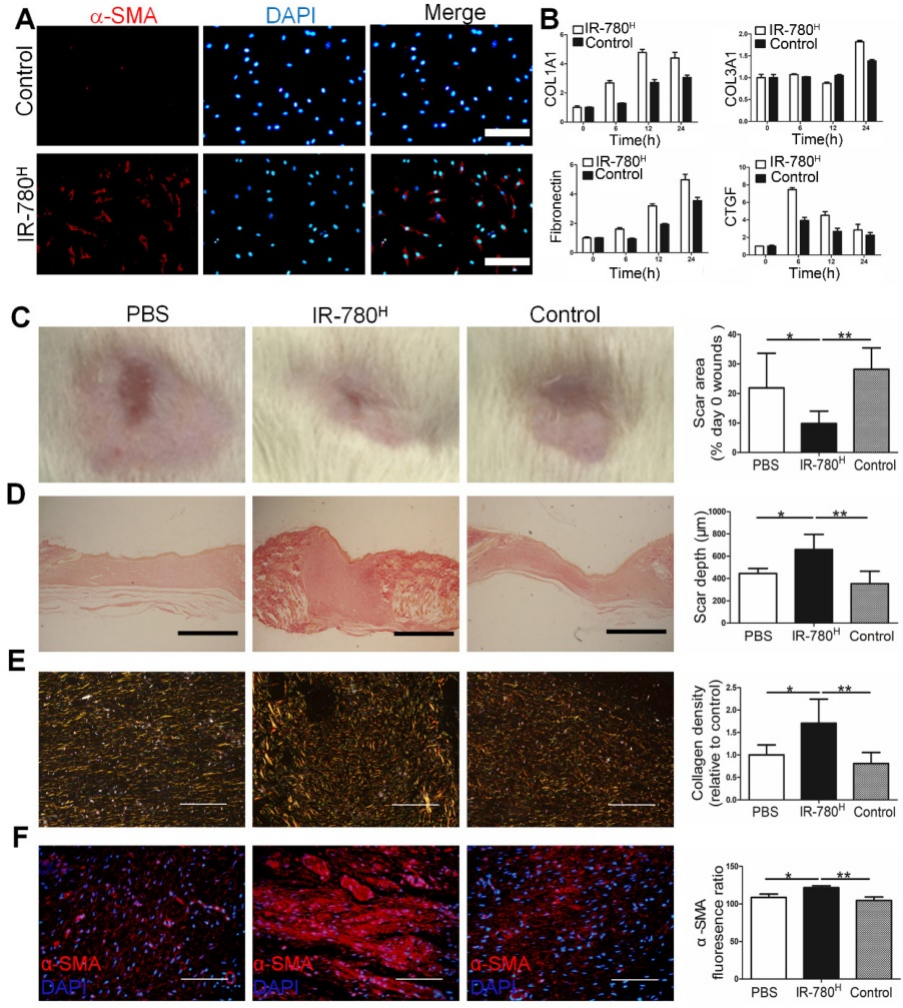
** TGF-β1 stimulation and transplantation of the IR-780^H^ fibroblasts into injured skin.**(A) Immunostaining of *TGF-β1* stimulated IR-780^H^ fibroblasts for *α-SMA*. Scale bar, 75µm. (B) real-time RT-PCR testing the expression of fibrogenic genes in IR-780^H^ fibroblasts following *TGF-β1* stimulation. Analysis of (C) the scar area, (D) scar depth, (E) ECM deposition and (F) expression of *α-SMA* in the healed wound tissues treated with systemically injecting of IR-780^H^ and control fibroblasts. Scale bar, 500 µm in (D), 100 µm in (E, F). Data are mean ± s.d.*,P<0.05;**,P<0.01.

As fundamental feature of cells, metabolism determines the cell fate and metabolic dysfunctions play important roles in the genesis and progress of fibrosis^21^, ^22^. Glycolysis, one of the most important metabolic pattern, is recently shown to be tightly related to fibrosis associated cells^17^, ^23^. Therefore, the fibrosis specific alterations of glycolysis related properties in fibrogenic cells have emerged as intriguing targets for fibrosis treatment. In this study, IR-780, a previously identified NIR dye for targeting glycolytic tumor cells, is further characterized to target a unique fibrogenic cell population in human and mouse fibroblasts. IR-780 is a small molecule emitting fluorescence in the NIR region and is preferentially localized in the mitochondria (Pearson's correlation coefficient is 0.886790) (**Figure 1A**). When labeling human or mouse fibroblasts with IR-780, few cells (≈5%) showed increased NIR-fluorescence intensity (**Figure 1B** and **Figure S1A**). The IR-780 enriched fibroblast population was subsequently isolated using flow cytometry (**Figure 1C**). We set the analytical threshold to 5% IR-780 fluorescent signal to sort for the IR-780-enriched fibroblasts, with these 5% most-highly fluorescent cells termed “IR-780^H^ fibroblasts” and the 5% most-weakly fluorescent cells termed “control fibroblasts.” Their fluorescence was confirmed under a NIR-fluorescence microscope (**Figure 1D and Figure S1B**). Morphologically, IR-780^H^ fibroblasts were long and spindle shaped, whereas control fibroblasts were triangular (**Figure 1E**). Additionally, IR-780^H^ fibroblasts demonstrated less proliferation and colony-forming ability (**Figure S1C, D and E**). Interestingly, fibrogenic gene expression was significantly higher in the IR-780^H^ fibroblasts than in the control fibroblasts (**Figure 1F to I**). Moreover, when stimulated by transforming growth factor β1 (*TGF-β1*), almost all of the IR-780^H^ fibroblasts were induced to express α-smooth muscle actin (*α-SMA*) after 12 h, whereas control fibroblasts showed only rare *α-SMA* expression (**Figure 2A**). Fibrogenic gene expression following *TGF-β1* stimulation was significantly higher in IR-780^H^ fibroblasts than in control fibroblasts (**Figure 2B and Figure S2A, B**).

### IR-780^H^ Fibroblasts Population Displayed High ECM Production *in vivo*

Next, we transplanted human IR-780^H^ fibroblasts and control fibroblasts into skin-wounded rats through the vena caudalis to test their relative contribution to scar formation. Wound-healing rate in the IR-780^H^ fibroblast transplantation group was faster than that in the control fibroblast transplantation group (**Figure S2C**). Moreover, wounds in the IR-780^H^ fibroblast transplantation group showed smaller scar area (**Figure 2C**), greater scar depth (**Figure 2D**), more extracellular matrix (ECM) deposition (**Figure 2E**) and increased α-SMA production (**Figure 2F**) than wounds in control fibroblast transplantation group and phosphate-buffered saline (PBS)-treated wounds. Next, we isolated IR-780^H^ and control neonatal fibroblasts from conditional fluorescent reporter R26^mTmG^ mice and transplanted them into the dorsal dermis of wild-type C57/BL mice. After 10 days, IR-780^H^ fibroblasts transplanted into the dermis had deposited more labeled ECM than control fibroblasts (**Figure S2D**). In addition, we co-transplanted B16 mouse melanoma cells with either IR-780^H^ or control neonatal fibroblasts from R26^mTmG^ mice into the dorsum of wild-type C57/BL mice. After 14 days, the IR-780^H^ fibroblast group produced significantly more labeled ECM in the tumor stroma than did the control fibroblasts (**Figure S2E**). This is consistent with earlier finding of a much greater fibrogenic potential of the IR-780^H^ fibroblast population than of the control population.

### IR-780 Targets Fibrogenic Fibroblast Population *in vivo*

**Figure 3:**
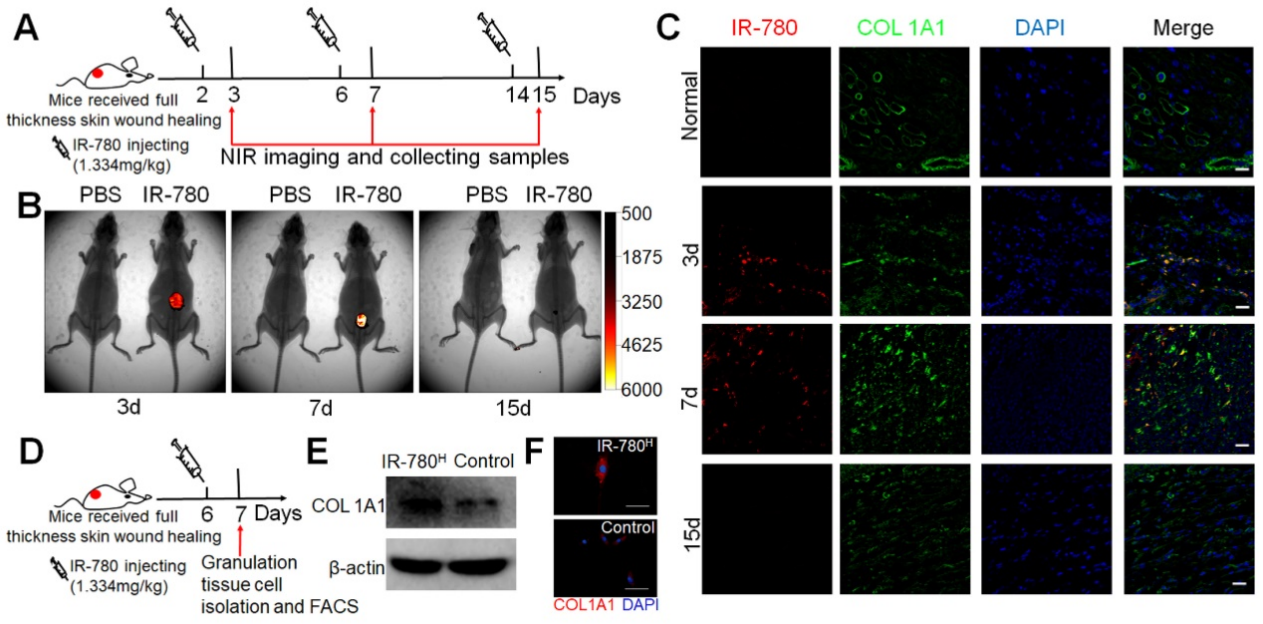
** IR-780 identifies fibrogenic fibroblasts *in vivo* .** (A) Schematic of the experimental strategy for IR-780 identification of fibrogenic cells following wounding. (B) the whole body NIR imaging of wounded mice following administrated IR-780. (C) Co-localization detection of IR-780 and *COL1A1* in wound tissues at indicated time points. Representative images of n = 3 samples/time point. Scale bar, 25 µm.(D) Schematic of the experimental strategy for isolation of fibrogenic cells in wound tissues by IR-780. (E) western bolt analyzing, (F) Immunostaining of isolated IR-780^H^ fibroblasts from wound tissues for *COL1A1*. Scale bar, 100 µm.

Further, we assessed whether IR-780 would target the fibrogenic subpopulation *in vivo* . Following the process described in Figure 3A, NIR-fluorescent images of the whole body and wound tissue showed the highly fluorescent contrast between wound sites and adjacent normal tissues at 3 and 7 days after wounding but not at 15 days (Figure 3B and Figure S3A), which was confirmed by immunofluorescence analysis. Further study showed most cells that accumulated plentiful IR-780 were COL1A1 positive (Figure 3C). Additionally, following *in vivo* IR-780 labeling, we isolated and directly sorted the IR-780^H^ from the control granulation tissue cells (Figure 3D) and confirmed that the IR-780^H^ granulation tissue cells had significantly higher rates of COL1A1 expression than control fibroblasts(Figure 3E, F). To exclude the risk of interference from non-fibrogenic cells with high IR-780 uptake, we firstly isolated granulation tissue cells by adherent culture and then incubated the adherent cells with IR-780 for further fluorescence-activated cell sorting (Figure S3B). The IR-780^H^ cells also exhibited higher COL1A1 expression than did the control cells (Figure S3C, D). In addition, we futher test the expression of previous reported profibrotic proteins including CD26^8^, CD29^24^. Results indicated that the expression percentages of CD29, CD26 were 93.5%, 57.9% respectively in IR-780^H^ fibroblasts (Figure S4A). Moreover, the surface expression levels of CD29 and CD26 were also significantly higher in IR-780^H^ fibroblasts than that in control fibroblasts (Figure S4B, C). Therefore, IR-780 could be used to identify the fibrogenic lineage during postnatal cutaneous wound healing *in vivo*.

### IR-780 Targets the Fibrogenic Fibroblast Population via HIF-1α/SLCO2A1 Pathway

**Figure 4:**
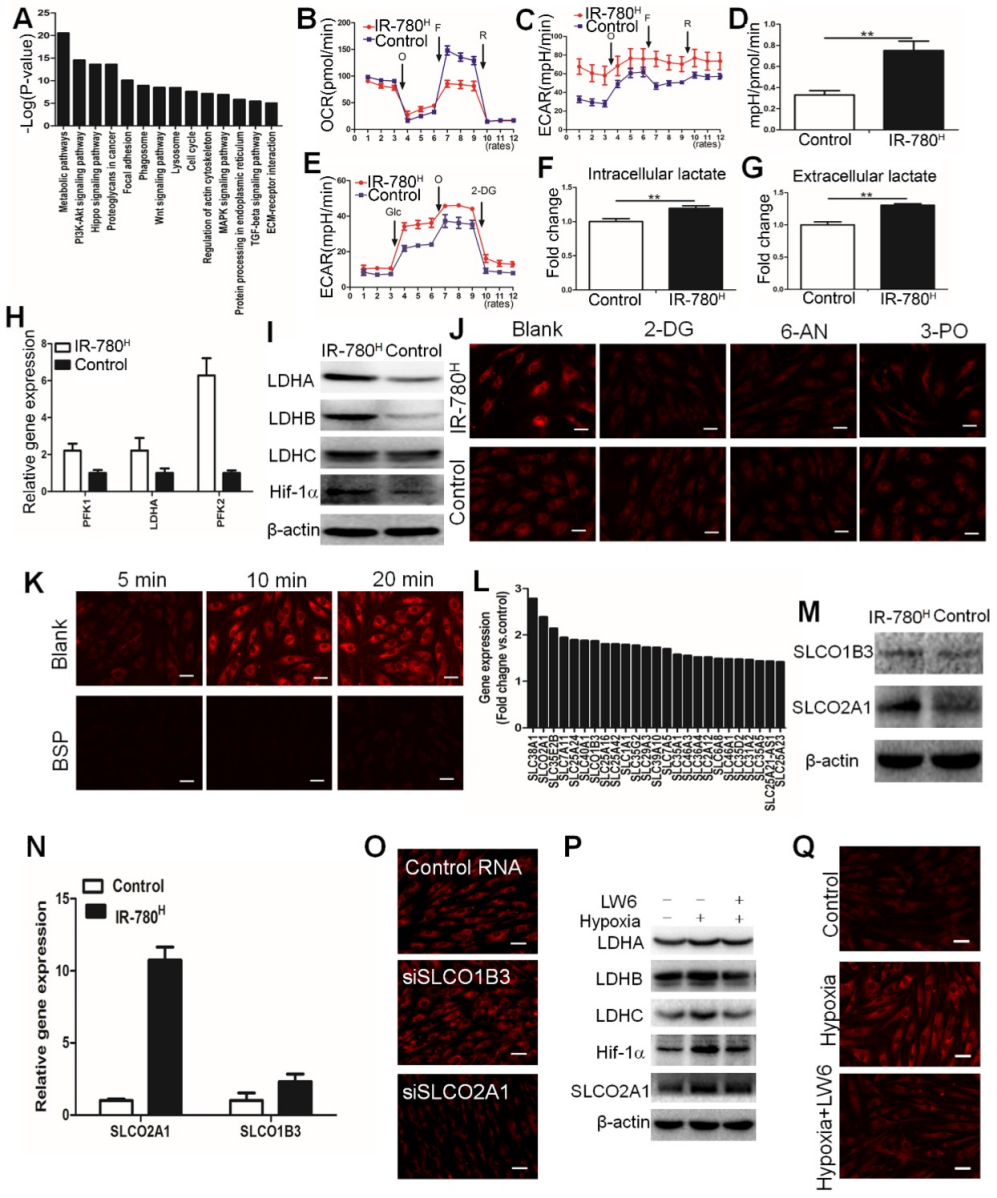
** Metabolic phonotype of IR-780^H^ fibroblasts.** (A) Up-regulated signaling pathways in IR-780^H^ fibroblasts compared to control fibroblasts. Real time (B) OCR, (C) ECAR and (D) mean ratios of ECAR:OCR of IR-780^H^ and control fibroblasts in mitochondrial stress testing.**,P<0.01. (E) Real time ECAR of IR-780^H^ and control fibroblasts in glycolysis stress testing. (F) Intracellular and (G) extracellular lactate levels in IR-780^H^ and control fibroblasts. (H) Realtime RT-PCR and (I) Western blot analysis of glycolytic enzyme and HIF-1α expression in IR-780^H^ and control fibroblasts. Testing the effects of glycolytic inhibitors (2-DG,6-AN,3PO) (J) and BSP (K) on the uptake of IR-780 in IR-780^H^ and control fibroblasts. (L) Up-regulated gene expression of members in solute carrier family in IR-780^H^ fibroblasts compared to control fibroblasts. Results of Western blot (M) and Real time PCR (N) of SLCO2A1 and SLCO1B3 in IR-780^H^ and control fibroblasts. (O) Testing the effects of siRNA of SLCO2A1 and SLCO1B3 on the uptake of IR-780 in fibroblasts. (P) The expression of LDHA, LDHB, LDHC, HIF-1αand SLCO2A1 and uptake of IR-780 (Q) in fibroblasts cultured in hypoxia ( 5% O2) with or without LW6.

Gene expression profile results of IR-780^H^ fibroblasts compared to control fibroblasts showed that metabolic pathway was the most differential upregulated pathway (**Figure 4A**). Moreover, IR-780^H^ fibroblasts exhibited decreased oxygen consumption rate (OCR) but increased extra-cellular differentiation rate (ECAR) and ECAR/OCR ratio (**Figure 4B to E**). Additionally, both intracellular and extracellular lactate concentrations were increased in IR-780^H^ fibroblasts (**Figure 4F, G**). Real-time PCR and western blot analysis also revealed upregulated glycolytic enzyme expression, including the phosphofructokinases *PFK1* and *PFK2* and the lactate dehydrogenases* LDHA*, *LDHB*, and *LDHC* (**Figure 4H, I**). To confirm whether hyperactive glycolysis is involved in the uptake of IR-780, we decreased the glycolytic rate using the glycolytic inhibitors 2-deoxy-D-glucose (2-DG), 6-aminonicotinamide (6-AN), and 3-(3-pyridinyl)-1-(4-pyridinyl)-2-propen-1-one (3-PO). Interestingly, 2-DG, 6-AN, and 3-PO significantly reduced the uptake of IR-780 in IR-780^H^ fibroblasts but not in control fibroblasts (**Figure 4J and Figure S5A**). Therefore, IR-780 could identify the glycolytic fibroblasts, with hyperactive glycolysis being an important property of the fibrogenic fibroblast population.

Next, we investigated whether cellular endocytosis was involved in the uptake of IR-780. The results acquired from pretreatments with various endocytotic inhibitors including chlorpromazine (clathrin inhibitor), amiloride (actin inhibitor), and methyl-β-cyclodextrin (MβCD, caveolae inhibitor) showed that cellular uptake of IR-780 was not mediated by endocytotic mechanism (**Figure S5B, C**). Reportedly, the organic-anion-transporting polypeptide (OATPs, also known as Solute Carrier Organic Anion (SLCO)) were one of the most important carriers of small molecule. Our previous work has showed that (also known as OATP1B3) played dominant role in the uptake of IR-780 in tumor cells^18^. Whether the OATPs especially SLCO1B3 mediates the transportation of IR-780 in fibrogenic fibroblast population is still unclear. Thus, we administrated the bromosulfophthalein (BSP, an OATPs inhibitor) to inhibit the OATPs, results showed that the IR-780 accumulation in fibroblasts were sharply decreased (**Figure 4K and Figure S5D**). Furthermore, according to the data of gene expression profile, the gene ontology analysis exhibited that the small molecule metabolic process was the most significantly upregulated process in IR-780^H^ fibroblasts compared to control fibroblasts (**Figure S5E**). About 25 members in solute carrier family (SLC) were upregulated (**Figure 4L**). Among them, SLCO2A1 and SLCO1B3 were belonged to OATPs family. Western blot confirmed that SLCO2A1 and SLCO1B3 were significantly upregulated in IR-780^H^ fibroblasts (**Figure 4M**), which were further verified by Realtime PCR (**Figure 4N**). To further investigate the key transporter of IR-780, we administrated SLCO2A1 and SLCO1B3 siRNA and found that the SLCO2A1 siRNA significantly affected the uptake of IR-780 (**Figure 4O and Figure S5F)**. These data demonstrated that the up-take of IR-780 in fibrogenic fibroblasts was mediated mainly by SLCO2A1. Further studies indicated that HIF-1a was up-regulated in the IR-780H fibroblasts and wound tissues (**Figure 4I and Figure S5G**). We then cultured the fibroblasts in 5% O2, the expression of HIF-1a, glycolic enzymes including LDHA, LDHB, LDHC and SLCO2A1 were significantly up-regulated. While LW6, a selectively HIF-1a inhibitor, decreased the expression of above genes (**Figure 4P and Figure S5H**). Previous studies reported that HIF-1α was shown to directly regulate the SLCO superfamily^25^, ^26^. Moreover, the uptake of IR-780 was increased in culture condition of 5% O2, while it was significantly decreased by LW6 (**Figure 4Q and Figure S5I**). Altogether, the above data suggested that HIF-1α was the key regulator of mediating the glycolytic phenotype and the SLCO2A1 based uptake of IR-780 in IR-780^H^ fibroblasts.

### IR-780 Based *in vitro* Photoinduced Cytotoxicity of The fibrogenic Fibroblast Population

**Figure 5:**
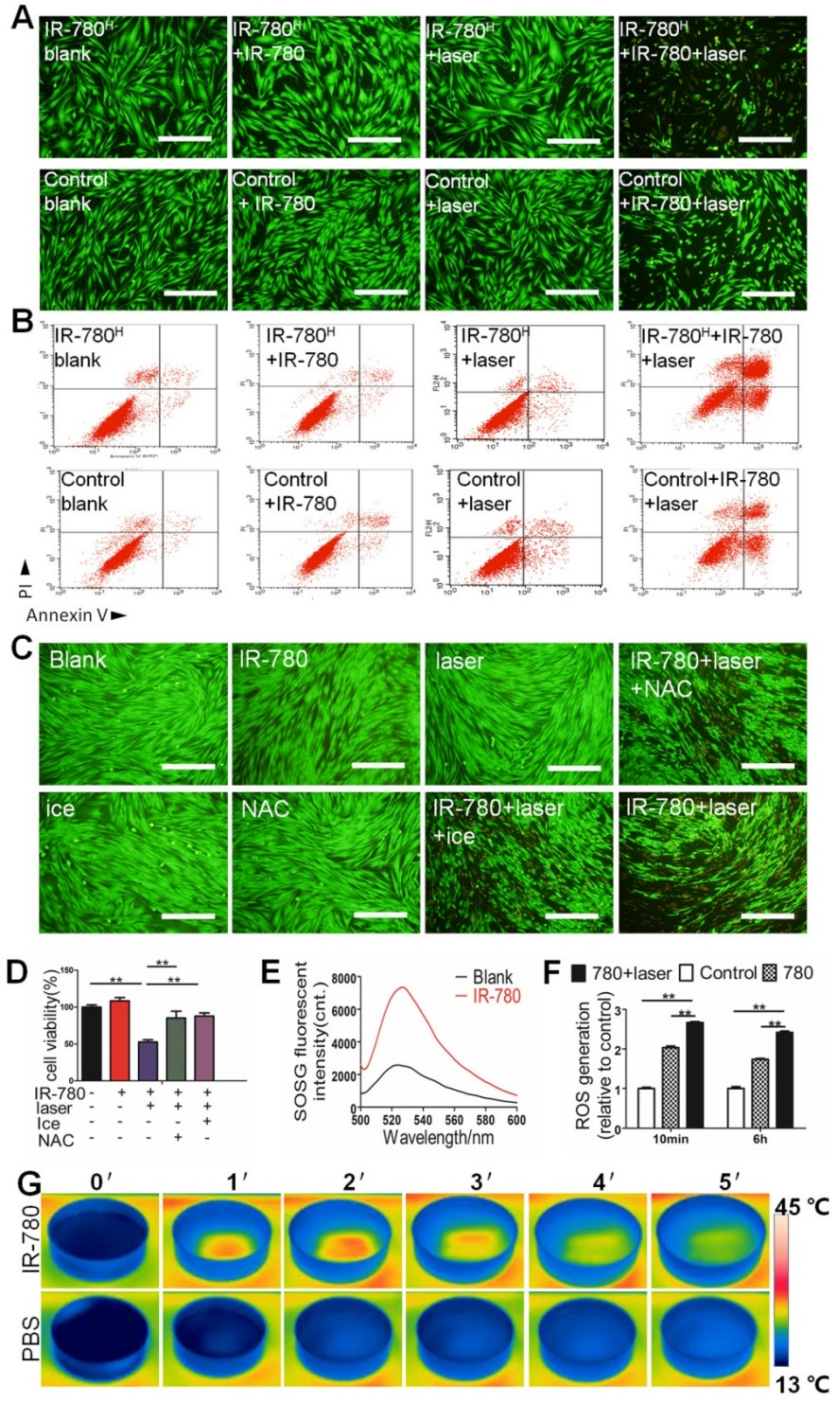
***In vitro* photoinduced cytotoxicity of IR-780^H^ fibroblast population.** (A) Calcein AM/ PI staining of IR-780^H^ and control fibroblasts exposed to 0.4 µM IR-780 with NIR laser of 1.25 W cm^-2^. Scale bar, 100 µm. (B) Apoptosis detection of human IR-780^H^ and control fibroblasts exposed to 0.4 µM IR-780 with NIR laser irradiation (808 nm, 1.25 W cm^-2^) by Flow Cytometry. (C) Calcein AM/ PI staining and (D) Cell viability tests of IR-780 treated primary fibroblasts with or without ice, NAC and NIR laser. **, P<0.01. (E)Singlet oxygen generation of IR-780 exposed to NIR laser, (F) the ROS generation of fibroblasts treated with 0.4 µM IR-780 and NIR laser. **, P<0.01. (G) Real time thermal images of IR-780 solution irradiated with NIR laser.

**Figure 6:**
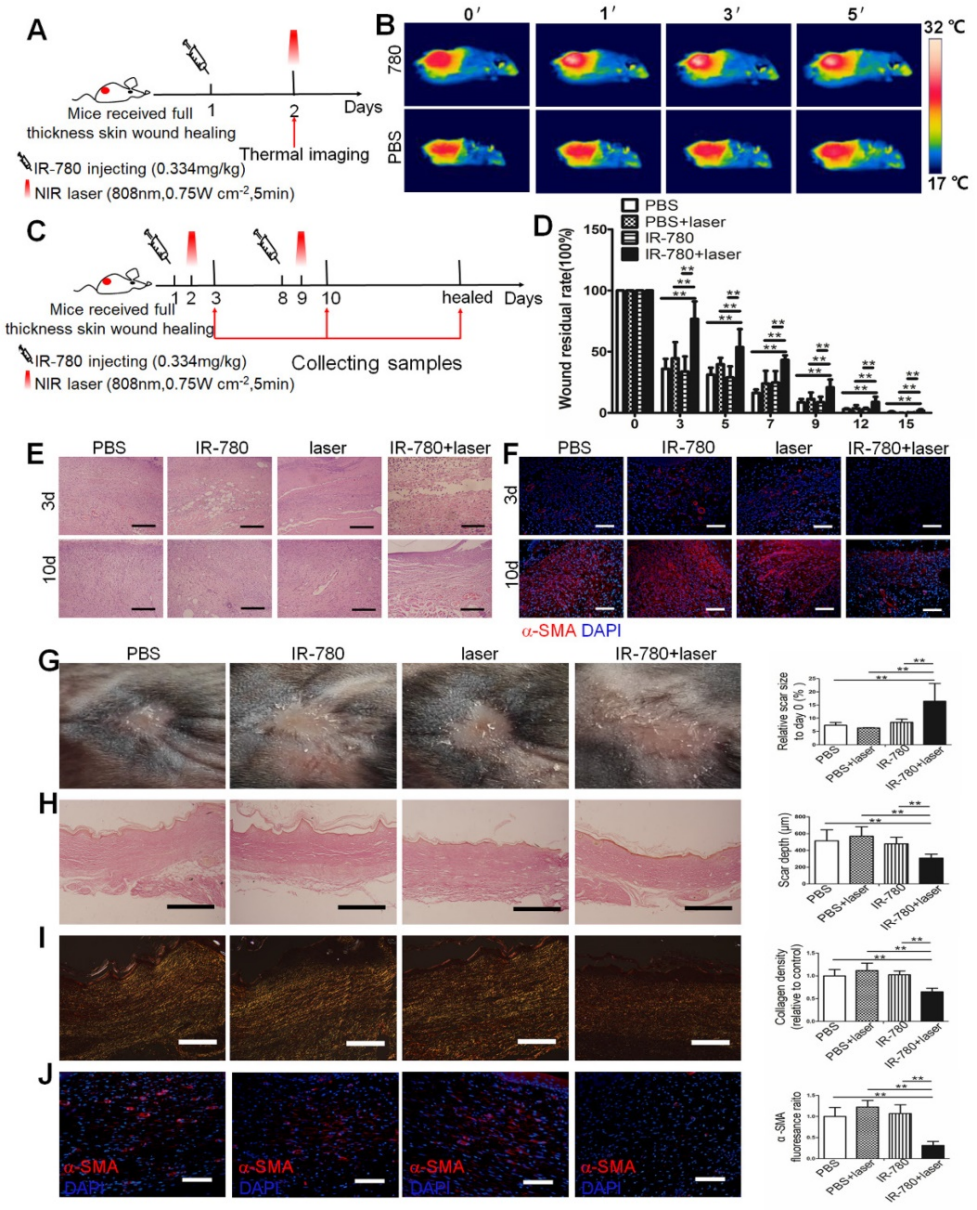
***in vivo* fibrogenic fibroblast targeting near-infrared phototherapy.** (A) Schematic of the experimental strategy for estimating the photothermal effect of IR-780 *in vivo* . (B) Real time thermal images of IR-780 treated wound tissues irradiated with NIR laser. (C) Schematic depicting the timing of IR-780 based photothermal, photodynamic therapy. (D) Wound healing rate with IR-780 +NIR laser treatment.**, P<0.01. (E) HE staining and (F) immunostaining of wound tissues treated with IR-780 + laser for α-SMA. Analysis of (G) the scar area, (H) scar depth, (I) ECM deposition and (J) expression of *α-SMA* in the healed wound tissues treated with IR-780. Scale bar, 500 µm in (C,H), 100 µm in (D,I, J). Data are mean ± s.d. **, P<0.01.

Reportedly, IR-780 and its derivatives possess photothermal (PTT) and photodynamic (PDT) properties^27^, ^28^ . Based on the characteristics that targeted the fibrogenic fibroblasts, we developed a fibrogenic cell ablating strategies by pretreating cells with IR-780, followed by topical laser irradiation. Results of *in vitro* cell viability, calcein AM/propidium iodide staining, and flow cytometry analysis (**Figure 5A, B and Figure S6**) showed sharply higher cell death incidences in IR-780^H^ fibroblasts than in control fibroblasts following IR-780 pretreatment and NIR-laser irradiation. To verify whether the therapeutic effect of IR-780 was contributed by synergetic PDT and PTT treatments, laser irradiation toward IR-780 was performed under ice incubation for single PDT treatment or pretreated with N -acetylcysteine (an effective scavenger for wiping out ROS) for single PTT treatment. Results of calcein AM/propidium iodide staining intuitively demonstrated that the photoinduced cytotoxicity of IR-780 was synergistically enhanced by PDT and PTT combinatorial treatment (**Figure 5C**). Cell viability further showed that 52.6% cell viability was detected in both PTT and PDT treatment, 85.2% cell viability in PTT treatment alone, and 87.8% cell viability in PDT treatment alone (**Figure 5D**). To confirm the PDT effect, the generation of singlet oxygen from IR-780 was determined to be significantly higher than that from blank water following irradiation (**Figure 5E**). Reactive oxygen species generation was also significantly higher in IR-780 pre-treated and irradiated fibroblasts than that in other groups (**Figure 5F**). To confirm the PTT effect, the temperature in IR-780 irradiation group rapidly increased above 40°C within the first 2 min irradiation. In contrast, the temperature in PBS irradiation group only accumulated to 15°C at the end of 5 min irradiation (**Figure 5G**). Results of *in vivo* detection of the PTT effect of IR-780 showed that the temperature in IR-780 irradiation group was significantly higher compared to the PBS group following 5 min irradiation (**Figure 6A, B**).

### IR-780 Based *in vivo* Fibrogenic Fibroblast Targeting NIR Phototherapy

Due to the intrinsic fibrogenic fibroblast population targeting property and the photoinduced cytotoxicity *in vitro,* we further investigated the potential of IR-780 based fibrogenic fibroblast population-targeted photoinduced therapeutic strategies. Following ablation of the fibrogenic fibroblast population by IR-780 combined with irradiation (**Figure 6C**), wound-healing rate was significantly decreased (**Figure 6D**), while the fibroblast number and *α-SMA* expression following phototherapy with IR-780 at 3 and 10 days were also reduced (**Figure 6E, F**). Conversely, cell death in cutaneous tissue following phototherapy with the IR-780 was sharply increased (**Figure S7**). Results of healed wounds revealed significantly enlarged scar area but greatly reduced scar depth, ECM deposition, and *α-SMA* expression after phototherapy with IR-780 (**Figure 6G to J**). Further, we did toxicity evaluation. We tested different concentrations of IR-780 on the toxicity of human fibroblasts and found that IR-780 > 10 μM exhibited killing effects (**Figure S8A**). Then histological analysis of different organs in mice receiving about 4 times of the IR-780 dosage administered in phototherapy showed no significant difference from control mice (**Figure S8B, C**). These data demonstrated that IR-780 based NIR phototherapy could effectively ablate the fibrogenic fibroblast population, and in turn sharply decreased the connective tissue deposition during wound healing. Our results indicated that the IR-780 based NIR phototherapy was a precision therapy because of the fibrogenic fibroblast population targeting property of the IR-780. In addition, none tested systemic toxicity of IR-780 would promise the IR-780 based NIR phototherapy as a potential therapeutic strategy for tissue fibrosis.

## Discussion

Fibroblasts, the predominant cells in mesenchymal compartment of organs, have been regarded as heterogeneous mesenchymal cell populations^6^, ^29^. Myofibroblasts have been considered the dominant cells for the fibrosis during the wound healing in adults^30^, ^31^. Though several studies have shown that the circulation derived cells such as bone marrow mesenchymal stem cells (BM-MSCs) and fibrocytes might be involved in the origins of myofibroblasts^32^, ^33^, the resident dermal fibroblasts have been proven to be the predominant contributor to myofibroblasts^34^. Recent studies further demonstrate that myofibroblasts are derived from not all of the dermal fibroblasts but specific dermal fibroblast subpopulations^6^, ^8^, ^10^, ^29^, ^35^. The relationship between fibroblast heterogeneity and the origins of the cutaneous myofibroblasts is still not fully characterized. Previous strategies to study the origin of myofibroblasts were mainly based on genetic lineage tracing, however, the markers used in different studies caused controversial results^6^, ^8^. Thus, marker-independent strategies to study the origin of the myofibroblasts are urgently needed. In this study, we identified a fibrogenic fibroblast subpopulation by metabolic functional features rather than genetic markers, which supplied a brand new methodology for distinguishing the dermal fibroblast subpopulations in fibrosis. Furthermore, this fibrogenic fibroblast subpopulation showed faster TGF-β response capability and much more ECM production than control fibroblasts, which suggested that this subpopulation would be a unique dermal cell population with prioritized transformation potential into myofibroblasts following injury.

**Figure 7:**
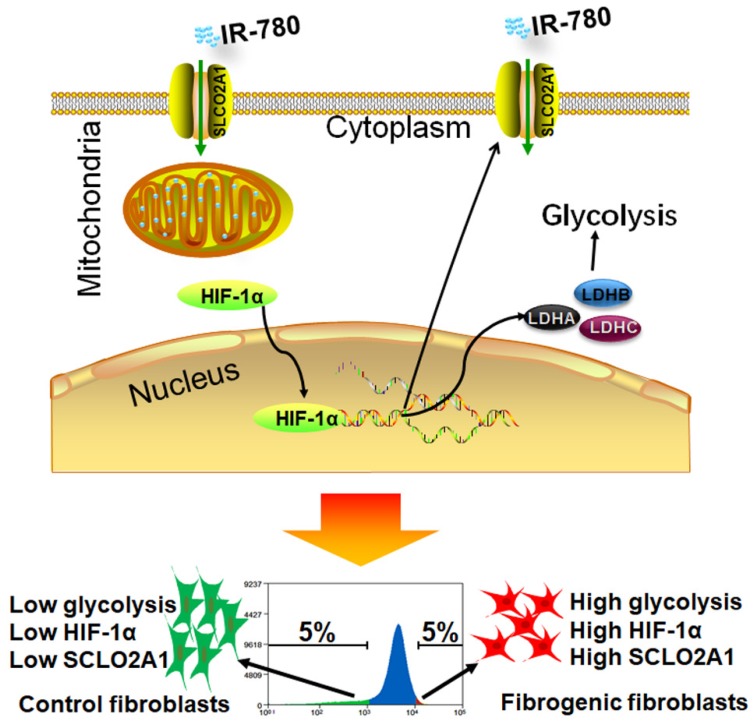
** Schematic illustration of identification of fibrogenic fibroblasts with IR-780.** In fibroblasts, HIF-1α regulates the expression of SLCO2A1, which mediates the uptake of IR-780. In another aspect, HIF-1α regulates the expression of glycolysis related enzymes including LDHA, LDHB, LDHC, which further mediates the glycolysis in fibroblasts. In fibrogenic fibroblasts, the expression of HIF-1α is high, which induces high glycolysis and high expression of SLCO2A1, and further mediates increased accumulation of IR-780.

Metabolism is the fundamental feature of cells, specific metabolic pathways are usually related to determined cell functions. Previous studies demonstrated that fibroblasts exhibited enhanced aerobic glycolysis in fibrosis related diseases such as skin fibrosis^13^, cancer^5^, laryngotracheal stenosis^14^, keloid^15^, pulmonary hypertension^16^, idiopathic pulmonary fibrosis^17^, et al. According to our previous study, the glycolytic levels in fibrogenic fibroblasts seemed to be lower than that in tumor cells^27^. Because of lacking effective strategies to identify metabolic activity in living cells, it is hard to investigate the relationship between cellular functions and metabolic activity in heterogeneous fibroblast populations. Despite several studies have developed methodologies for identifying oxidative phosphorylation (mitochondrial activity); glycolytic activity identification was urgently needed. Here, we reported a methodology to identify the glycolytic activity with a small molecule in living cells (**Figure 7**). By using this methodology, we found that hyperactive glycolysis fibroblast population was the primary contributor to ECM deposition during cutaneous wounding, and cancer stroma formation, which suggested glycolytic diversity was tightly related to fibroblast heterogeneity. Hyperactive glycolysis might be the functional phenotype of fibrogenic population. However, the underline mechanism linking the metabolic pathways and fibroblast heterogeneity still needs further studies.

Till now, the main treatment strategies for fibrosis were focus on signaling pathways such as typical *TGF-β* pathway, *β-catenin* pathway et al^36^, ^37^, however, these pathways also act in many non-fibrogenic cells, in which they might be responsible for other basic cell functions. Despite having achieved some clinical effects, systemic toxicity associated with the inhibition of such signaling pathways may limit the maximal tolerated dose of these drugs therapies^22^. Recently, several studies have revealed that specific fibroblast population would be responsible for ECM deposition in different injury conditions^9^-^12^, ^38^, these fibroblast population targeting therapeutic strategies wound be great potential, of which specific cell surface markers based therapy attracted great interests. However, it still not reaches consensus about the specific markers for fibrogenic fibroblast subpopulation. It might be a long way to carry out the surface markers dependent therapy. Our study supplied brand new therapeutic strategies for fibrosis based on a fibrogeinc fibroblast targeting NIR fluorescent molecule IR-780. Furthermore, IR-780 has the properties of photothermal and photodynamic effects, which together with its easily synthesis and preparation promised this kind of small molecules based precision therapy as a potential therapeutic strategy for fibrosis.

## Supplementary Material

Supplementary figures and tables.

## Abbreviations

NIR: near-infrared
ECM: extracellular matrix
PTT: photothermal
PDT: photodynamic
RT: room temperature
2-DG: 2-Deoxy-D-glucose
6-AN: 6-aminonicotinamide
3PO: 3-(3-Pyridinyl)-1-(4-pyridinyl)-2-propen-1-one
OATPs: organic-anion-transporting polypeptides
BSP: sulfobromophthalein disodium salt hydrate
*FACS*: Fluorescence activated cell sorting
ECAR: extracellular acidification rate
OCR: oxygen consumption rate
FCCP: carbonyl cyanide 4-(trifluoromethoxy) phenylhydrazone
TUNEL: Terminal deoxynucleotidyl transferase-mediated deoxyuridine triphosphate nick end labeling
*TGF-β1*: transforming growth factor β1
*α-SMA*: α-smooth muscle actin
SLCO: Solute Carrier Organic Anion
NAC: N -acetylcysteine

## References

[1] Henderson NC, Arnold TD, Katamura Y, Giacomini MM, Rodriguez JD, McCarty JH (2013). Targeting of alphav integrin identifies a core molecular pathway that regulates fibrosis in several organs. Nat Med.

[2] Sorrell JM, Caplan AI (2009). Fibroblasts-a diverse population at the center of it all. Int Rev Cell Mol Biol.

[3] Finnerty CC, Jeschke MG, Branski LK, Barret JP, Dziewulski P, Herndon DN (2016). Hypertrophic scarring: the greatest unmet challenge after burn injury. Lancet.

[4] Gurtner GC, Werner S, Barrandon Y, Longaker MT (2008). Wound repair and regeneration. Nature.

[5] Kalluri R (2016). The biology and function of fibroblasts in cancer. Nat Rev Cancer.

[6] Driskell RR, Lichtenberger BM, Hoste E, Kretzschmar K, Simons BD, Charalambous M (2013). Distinct fibroblast lineages determine dermal architecture in skin development and repair. Nature.

[7] Driskell RR, Watt FM (2015). Understanding fibroblast heterogeneity in the skin. Trends Cell Biol.

[8] Rinkevich Y, Walmsley GG, Hu MS, Maan ZN, Newman AM, Drukker M (2015). Skin fibrosis. Identification and isolation of a dermal lineage with intrinsic fibrogenic potential. Science.

[9] LeBleu VS, Taduri G, O'Connell J, Teng Y, Cooke VG, Woda C (2013). Origin and function of myofibroblasts in kidney fibrosis. Nat Med.

[10] Dulauroy S, Di Carlo SE, Langa F, Eberl G, Peduto L (2012). Lineage tracing and genetic ablation of ADAM12(+) perivascular cells identify a major source of profibrotic cells during acute tissue injury. Nat Med.

[11] Iwaisako K, Jiang C, Zhang M, Cong M, Moore-Morris TJ, Park TJ (2014). Origin of myofibroblasts in the fibrotic liver in mice. Proc Natl Acad Sci U S A.

[12] Kramann R, Schneider RK, DiRocco DP, Machado F, Fleig S, Bondzie PA (2015). Perivascular Gli1+ progenitors are key contributors to injury-induced organ fibrosis. Cell Stem Cell.

[13] Zhao X, Psarianos P, Ghoraie LS, Yip K, Goldstein D, Gilbert R (2019). Metabolic regulation of dermal fibroblasts contributes to skin extracellular matrix homeostasis and fibrosis. Nat Metab.

[14] Ma G, Samad I, Motz K, Yin LX, Duvvuri MV, Ding D (2017). Metabolic variations in normal and fibrotic human laryngotracheal-derived fibroblasts: A Warburg-like effect. Laryngoscope.

[15] Li Q, Qin Z, Nie F, Bi H, Zhao R, Pan B (2018). Metabolic reprogramming in keloid fibroblasts: Aerobic glycolysis and a novel therapeutic strategy. Biochem Biophys Res Commun.

[16] Zhang H, Wang D, Li M, Plecita-Hlavata L, D'Alessandro A, Tauber J (2017). Metabolic and Proliferative State of Vascular Adventitial Fibroblasts in Pulmonary Hypertension Is Regulated Through a MicroRNA-124/PTBP1 (Polypyrimidine Tract Binding Protein 1)/Pyruvate Kinase Muscle Axis. Circulation.

[17] Xie N, Tan Z, Banerjee S, Cui H, Ge J, Liu RM (2015). Glycolytic Reprogramming in Myofibroblast Differentiation and Lung Fibrosis. Am J Respir Crit Care Med.

[18] Zhang E, Luo S, Tan X, Shi C (2014). Mechanistic study of IR-780 dye as a potential tumor targeting and drug delivery agent. Biomaterials.

[19] Chen Z, Dai T, Chen X, Tan L, Shi C (2015). Activation and regulation of the granulation tissue derived cells with stemness-related properties. Stem Cell Res Ther.

[20] Chen Z, Wang X, Jin T, Wang Y, Hong CS, Tan L (2017). Increase in the radioresistance of normal skin fibroblasts but not tumor cells by mechanical injury. Cell Death Dis.

[21] Kusminski CM, Bickel PE, Scherer PE (2016). Targeting adipose tissue in the treatment of obesity-associated diabetes. Nat Rev Drug Discov.

[22] Breyer MD, Susztak K (2016). The next generation of therapeutics for chronic kidney disease. Nat Rev Drug Discov.

[23] Chen Y, Choi SS, Michelotti GA, Chan IS, Swiderska-Syn M, Karaca GF (2012). Hedgehog controls hepatic stellate cell fate by regulating metabolism. Gastroenterology.

[24] Shook BA, Wasko RR, Rivera-Gonzalez GC, Salazar-Gatzimas E, Lopez-Giraldez F, Dash BC (2018). Myofibroblast proliferation and heterogeneity are supported by macrophages during skin repair.

[25] Wu JB, Shao C, Li X, Shi C, Li Q, Hu P (2014). Near-infrared fluorescence imaging of cancer mediated by tumor hypoxia and HIF1alpha/OATPs signaling axis. Biomaterials.

[26] Wang Y, Liao X, Sun J, Yi B, Luo S, Liu T (1700). Characterization of HIF-1α/Glycolysis Hyperactive Cell Population via Small-Molecule-Based Imaging of Mitochondrial Transporter Activity. Adv Sci.

[27] Luo S, Tan X, Fang S, Wang Y, Liu T, Wang X (2016). Mitochondria-Targeted Small-Molecule Fluorophores for Dual Modal Cancer Phototherapy. Adv Funct Mater.

[28] Xia F, Niu J, Hong Y, Li C, Cao W, Wang L (2019). Matrix metallopeptidase 2 targeted delivery of gold nanostars decorated with IR-780 iodide for dual-modal imaging and enhanced photothermal/photodynamic therapy. Acta Biomater.

[29] Varkey M, Ding J, Tredget EE (2011). Differential collagen-glycosaminoglycan matrix remodeling by superficial and deep dermal fibroblasts: potential therapeutic targets for hypertrophic scar. Biomaterials.

[30] Darby IA, Zakuan N, Billet F, Desmouliere A (2016). The myofibroblast, a key cell in normal and pathological tissue repair. Cell Mol Life Sci.

[31] Tan J, Wu J (2017). Current progress in understanding the molecular pathogenesis of burn scar contracture. Burns Trauma.

[32] Wu Y, Zhao RC, Tredget EE (2010). Concise review: bone marrow-derived stem/progenitor cells in cutaneous repair and regeneration. Stem Cells.

[33] Fan X, Liang HP (2010). Circulating fibrocytes: a potent cell population in antigen-presenting and wound healing. Chin J Traumatol.

[34] Barisic-Dujmovic T, Boban I, Clark SH (2010). Fibroblasts/myofibroblasts that participate in cutaneous wound healing are not derived from circulating progenitor cells. J Cell Physiol.

[35] Marangoni RG, Korman BD, Wei J, Wood TA, Graham LV, Whitfield ML (2015). Myofibroblasts in murine cutaneous fibrosis originate from adiponectin-positive intradermal progenitors. Arthritis Rheumatol.

[36] Nanthakumar CB, Hatley RJ, Lemma S, Gauldie J, Marshall RP, Macdonald SJ (2015). Dissecting fibrosis: therapeutic insights from the small-molecule toolbox. Nat Rev Drug Discov.

[37] Gourdie RG, Dimmeler S, Kohl P (2016). Novel therapeutic strategies targeting fibroblasts and fibrosis in heart disease. Nat Rev Drug Discov.

[38] Marcelin G, Ferreira A, Liu Y, Atlan M, Aron-Wisnewsky J, Pelloux V (2017). A PDGFRalpha-Mediated Switch toward CD9high Adipocyte Progenitors Controls Obesity-Induced Adipose Tissue Fibrosis. Cell Metab.

