# Supplementary material for: Fibrogenic fibroblast-selective near-infrared phototherapy to control scarring
Source: Theranostics. 2019 Sep 19;9(23):6797–808. doi: 10.7150/thno.36375 (PMC6815952; doi:10.7150/thno.36375)
Supplement: Supplementary file 1 — Supplementary figures and tables. [file thnov09p6797s1.pdf]

Fibrogenic fibroblast-selective near-infrared phototherapy to control scarring

Zelin Chen[1], Ziwen Wang[1], Taotao Jin[1], Gufang Shen[1], Yu Wang[1], Xu Tan[1], Yibo Gan[1], Fan Yang[1], Yunsheng Liu[1], Chunji Huang[2], Yixin Zhang[3]*, Xiaobing Fu[4]*, Chunmeng Shi[1]*

Supplemental information

Materials and Methods

Growth curve assay FACS-sorted IR-780^H or control human fibroblasts were seeded in 96-well plates (2×10^3 cells/well) with medium replaced every 3 days. Cell proliferation was daily assessed for 7 days by adding 10 μL CCK-8 (Dojindo) reagents into each well. The plate was incubated at 37℃ for 2 hours. Then absorbance at 450 nm was measured using a microplate reader (Thermo Scientific).

Surface marker detection by Flow Cytometry *Mice* were intraperitoneally injected with IR-780 (1.334mg/kg) at 6 days post wounding. 24 hours later, granulation tissue cells were isolated as described in 'cell isolation and culture' section. Granulation tissue cells then were incubated 40min with surface marker antibodies. After three washes with PBS. Co-expression of surface markers in control fibroblasts and IR-780^H fibroblasts were detected by BD FACSAria II.

Real-time cell metabolism assay XFe Extracellular Flux Analyzer (Seahorse Bioscience) was used for real-time analysis of extracellular acidification rate (ECAR) and oxygen consumption rate (OCR). FACS-sorted IR-780^H or control fibroblasts (2×10^4 cells/well) were seeded in Seahorse XF-8 cell culture microplates. To test the mitochondrial stress, cells were sequentially treated with 1μM oligomycin (Oligo), 2 μM carbonyl cyanide 4-(trifluoromethoxy) phenylhydrazone (FCCP) and 1μM

Rotenone. To test the glycolysis stress, cells were sequentially treated with 10 mM Glucose, 1 μM Oligo and 50 mM 2-DG. Real-time ECAR and OCR were recorded according to the manufacturer's manual.

Intracellular and extracellular lactate assays FACS-sorted IR-780^H or control fibroblasts were seeded in a 24-well plates and cultured for 48 hours. The culture medium and cells were collected respectively. Lactate in the medium and cell lysates were detected using a lactate assay kit (Nanjing Jiancheng engineering # A019-2) according to the manufacturer's manual.

RNA sequencing and quantitative real time *PCR*Total RNA was extracted using RNAiso Plus (TaKaRa). RNA sequencing and real-time RT PCR analysis of FACS-sorted human IR-780^H and control fibroblasts were performed by Gminix Biotechnology company(Shanghai, China). Quantitative PCR was conducted using a SYBR Premix Ex TaqII (TliRnaseH Plus) real-time PCR kit (TaKaRa) according to the manufacturer's protocol. Relative expression was normalized to the expression of *β-actin*.

Western blot Total proteins from fibroblasts were extracted using cell lysis buffer (Beyotime) supplemented with proteinase inhibitor cocktail (Roche). After vortexing and centrifugation at 12000 ×g for 10 min at 4°C, the supernatant was collected and protein was quantitated using a BCA protein assay kit (Beyotime). Equal amounts of protein for each sample were subjected to 12% SDS-PAGE and transferred to polyvinylidene difluoride membranes (Millipore). Membranes were blocked in blocking buffer (Beyotime) for 1 hour before application of primary antibodies 4°C

overnight. HRP-conjugated goat anti-mouse or anti-rabbit secondary antibody (1:1,000; Beyotime) was applied for 2 h at RT. Blots were visualized using chemiluminescence (Bio-Rad)

Immunofluorescence and immunohistochemistry IR-780 labeled wound tissue cryosections (10 μm) for immunofluorescence were stained as follows. Slides were washed thrice for 10 min at RT in PBS and then permeabilized with 0.1% Triton X100 for 5 min at RT and blocked in blocking buffer (PBS + 5% donkey serum (Beyotime) for 1 h at RT and then incubated overnight with primary antibodies diluted in PBS in a humidified chamber. The following day, slides were washed thrice in PBS for 10 min at RT and stained with Alexa Fluor 488 or cy3–conjugated secondary antibodies (diluted in PBS) for 30 min at 37 ℃. After three washes in PBS, slides were stained with DAPI at RT for 3min and mounted with Prolong Diamond antifade Reagent. The protocols of cell climbing slices for immunofluorescence were the same as the cryosections, except the permeation process: slides were permeabilized with 0.1% Triton X100 for 30 min at RT. The protocols of dewaxed paraffin sections (4 μm) for immunofluorescence were the same as cryosections, except the permeation process: slides were incubated in 0.1M citric acid > 95 ℃ for 20 min. Dewaxed paraffin sections (4 μm) for immunohistochemistry were washed thrice for 10 min at RT in PBS and incubated in 0.1M citric acid > 95 ℃ for 20 min. The slides were then blocked and incubated overnight with primary antibodies. The following day, slides were washed thrice in PBS for 10 min at RT and incubated with HRP–conjugated secondary antibodies (diluted in PBS) for 30

min at 37 ℃. After three washes with PBS, slides were incubated with DAB and co-stained with hematoxylin. All the sections were captured with Leica confocal microscrope or Olympus fluorescence microscope and processed with image J.

Cell transplantation

Human fibroblasts transplantation to rat wound model After dorsal skin wounding, 18 rats were randomly divided into three groups including IR-780^H group, control cell group and PBS group. Rats of IR-780^H group received 2×10^6 IR-780^H human fibroblasts in 0.2 ml PBS injected from tail vein. Rats of control cell group received 2×10^6 control human fibroblasts. Rats in PBS group were performed with 0.2 ml PBS. Wound tissues were harvested after wound healed (about 15 days after wounding). The wound scar area, scar depth, the ECM deposition and the expression of *α-SMA* were analyzed.

Melanoma transplantation The B16-F10 mouse melanoma cell line (ATCC) and was passaged twice in culture and injected inter-dermally into the dorsal skin of each C57/BL mouse with FACS-sorted IR-780^H or control neonatal ROSA26^{mTmG} fibroblasts (melanoma cells:fibroblasts as 5.0 ×10^4:1.0 ×10^6 cells per injection). After 14 days, tumors were harvested for histology.

Subcutaneous transplantation 1.0×10^6 FACS-sorted IR-780^H or control neonatal ROSA26^{mTmG} fibroblasts were transplanted into the dorsal dermis of C57/BL mice[8]. 10 days latter, all skin tissues were harvested for histology.

Sirius red staining Paraffin-embedded sections were dehydrated and stained in Sirius red solution for 1 hour, then mounted with Poly-Mount Xylene. Polaroid lens were

used for images taken under a microscope. The collagen density of each image was analyzed by Image J.

Cell viability analysis Fibroblasts were seeded in the 96-wells plates (1×10^4 cells/ well) and cultured overnight. Cells were subjected to different treatments before testing the cell viability : 1) To test the directly killing effect of IR-780, primary human fibroblasts were incubated with different concentrations of IR-780 (0.5, 1,2,4,6,8,10,15,20 μM) at 37°C for 20 min. 2) to test the photodynamic and photothermal effect of IR-780, FACS-sorted IR-780^H or control cutaneous fibroblasts were incubated with different concentrations of IR-780 (0.2,0.4,0.6,0.8,1μM) at 37°C for 10 min. After thrice washes, cells were subjected to NIR light irradiation using an infrared thermal camera (808 nm, 0.25, 1.25, 5.01 W cm^{-2}) for 5 min. 3) to study the photodynamic and photothermal mechanism of IR-780, primary fibroblasts were treated with or without NAC(5 mM, Sigma) for 24 hours and then incubated with IR-780 (0.4 μM) for 10 min. After thrice washes, cells incubated with or without ice were subjected to NIR light irradiation (1.25 W cm^{-2}) for 5 min. 24 hours after different treatments, cell viability was assessed using theCCK-8 assay (as described in section "Growth curve assay")

Calcein AM/(Propidium Iodide) PI staining Fibroblasts were seeded in the 6-wells plates (5×10^5 cells/ well) and cultured overnight. Cells were subjected to different treatments before testing the cell survival with Calcein AM/PI staining. To test the photodynamic and photothermal effect of IR-780, FACS-sorted IR-780^H or control human fibroblasts were incubated with IR-780 (0.4 μM) at 37 °C for 10 min. After

thrice washes, cells were irradiated with NIR laser light (808 nm, 1.25 W cm^{-2}) for 5 min. 6 hours after different treatments, cells were stained with Calcein AM(2ug/ml)+PI(15ug/ml) in PBS for 20 min at 37°C in the dark. After thrice washes, Fluorescent images were captured by Olympus fluorescence microscope and processed with image J.

Apoptosis detection by Flow Cytometry FACS-sorted IR-780^H or control human fibroblasts were seeded in the 6-wells plates (5×10^5 cells/ well) and cultured overnight. Cells were subjected to different treatments as described in section 'Calcein AM/(Propidium Iodide) PI staining'. 6 hours after different treatments, cells were harvested, washed twice with cold PBS, resuspended in Annexin-V binding buffer (10 mM HEPES, 150 mM NaCl, 5 mM KCl, 1 mM MgCl2, and 1.8 mM CaCl2, Ph 7.4) with FITC-conjugated Annexin-V (5ul vol. per test, BD Biosciences, 51-65874X) and PI (50 μg/ml, Sigma, P4170) for 20 minutes at RT in the dark. Samples were analyzed by BD Accuri™ C6 Flow Cytometer.

ROS detection by Flow Cytometry Primary human fibroblasts were seeded in the 6-wells plates (5×10^5 cells/ well) and cultured overnight. Cells were subjected to different treatments.1) To test the IR-780 induced ROS generation, cells were incubated with IR-780 (1μM) at 37°C for 20 min; 2) to test the photodynamic effect of IR-780 induced ROS generation, cells were incubated with IR-780 (0.4 1μM) at 37°C for 20 min, After thrice washes, cells were irradiated with NIR laser light (808 nm, 1.25 W cm^{-2}) for 5 min. Cells at different time points post treatments were incubated with H_2DCF-DA (intracellular ROS detection, 10μM, Sigma-Aldrich) andMitoSOX

Red (mitochondrial superoxide detection, 5 μM, Molecular Probes) for 20 min at 37°C. Cells were then collected. Mean fluorescence intensity of H_2DCF-DA (ex/em:488/525 nm) and MitoSOX Red (ex/em: 510/580 nm) were captured by BD Accuri™ C6 Flow Cytometer.

Single Oxygen Detection SOSG (Molecular Probes) was employed to evaluate the generation of singlet oxygen of IR-780. IR-780(10 μM) and SOSG (1.5 μM) in water containing 2% methanol were mixed and irradiated for 5 min (808 nm, 1.25 W cm−2). The fluorescence intensity of the irradiated solution was promptly determined by NIR fluorescence spectrometer. The emission peak at 530 nm of SOSG was afforded by exciting with a light resource of 494 nm wavelength and quantified for the singlet oxygen generation.

In vitro and vivo assessment of PTT property For in vitro detection, 2 ml IR-780 (10 μM) in PBS was filled in a 30 mm dish and irradiated with NIR laser light (808 nm, 1.25 W cm^{-2}) for 5 min. The real-time temperature change was imaged by the infrared thermal camera (Ti32, Fluke, USA). For in vivo detection, mice at 2 days after wounding were received intraperitoneal injection of IR-780 (0.334 mg/kg) in 0.2 ml PBS. 24 hours latter, the real-time temperature change of mice was imaged by the infrared thermal camera (Ti32, Fluke, USA) when the whole wound tissues were exposed to the continuous NIR laser beam (808 nm, 0.75 W/cm^2, 5 min).

In vivo therapeutic effect of IR-780 For photodynamic and photothermal therapeutic effects, mice were intraperitoneally injected with IR-780 (0.334mg/kg) at 1, 8 days post wounding. 24 hours latter, wound tissues were irradiated with NIR laser light

(808 nm, 1.25 W cm^{-2}) for 5 min. Wound samples were collected for histology at 3, 10 days and when the wounds healed.

TUNEL staining Terminal deoxynucleotidyl transferase–mediated deoxyuridine triphosphate nick end labeling (TUNEL) staining was performed using in situ cell death detection kit (Roche) according to the manufacturer's protocol. Analysis was performed by counting positive cells per high-power field.

RNA Interference siRNA targeting SLCO2A1,SLCO1B3 and nonspecifc siRNA were used to transiently knockdown these genes expression. The sequences of siRNA were as listed in 'The sequences of siRNA'. The siRNA were transfection using lipofectamine 3000 (Invitrogen) according to the manufactures' protocol.

Primers and Antibodies

Human primers *COL 1A1*, forward, 5′- GTGCGATGACGTGATCTGTGA -3′; *COL 1A1,* reverse,5′- CGGTGGTTTCTTGGTCGGT -3′; *COL3 A1*, forward, 5′- TTGAAGGAGGATGTTCCCATCT -3′; *COL 3A1,* reverse,5′- ACAGACACATATTTGGCATGGTT -3′; *CTGF*, forward, 5′- TCCACCCGGGTTACCAATG -3′; *CTGF,* reverse,5′- CAGGCGGCTCTGCTTCTCTA -3′; *TGF-β1*, forward, 5′- GGGAAATTGAGGGCTTTCG -3′; *TGF-β1,* reverse,5′- AGTGTGTTATCCCTGCTGTCACA -3′; *Fibronectin*, forward, 5′- CCAATTCCTTGCTGGTATCATG -3′; *Fibronectin,*reverse,5′- TCATACTTGATGATGTAGCCGGTAA -3′; *α -SMA*, forward, 5′- GCTCACGGAGGCACCCCTGAA -3′; *α -SMA,* reverse,5′-

TCCAGAGTCCAGCAGATG -3′;*Versican*, forward, 5′-GCAGCTGAACGGGAATGC -3′; *Versican,* reverse,5′-CGTGAGACAGGATGCTTGTGA -3′; *PFK1*, forward, 5′-GGTGCCCGTGTCTTCTTTGT -3′; *PFK1*, reverse,5′-AAGCATCATCGAAACGCTCTC -3′; *PFK2*, forward, 5′-ATTGCGGTTTTCGATGCCAC -3′; *PFK2,* reverse,5′-GCCACAACTGTAGGGTCGT -3′; *LDHA*, forward, 5′-ATGGCAACTCTAAAGGATCAGC -3′; *LDHA,* reverse,5′-CCAACCCCAACAACTGTAATCT -3′; *β -actin*, forward, 5′-CATGTACGTTGCTATCCAGGC -3′; *β -actin,* reverse,5′-CTCCTTAATGTCACGCACGAT -3′.

Antibodies *COL1A1* (Abcam, ab34710); *Fibronectin (*Abcam, ab2413); *p-smad3* (Abcam, ab52903); *SP1* (Abcam, ab13370); *TGF-β1* (Abcam, ab92486); *CTGF* (Abcam, ab6992); *LDHA* (Proteintech, 19987-1-AP); *LDHB* (Proteintech, 14824-1-AP); *LDHC* (Proteintech, 19989-1-AP); *β-actin* (Beyotime, AA128); *α-SMA* (Abcam, ab5694); *RFP* (Abcam, ab62341); *HIF-1 α* (Proteintech, 20960-1-AP); *SLCO1B3* (Proteintech, 66381-1-Ig); *SLCO2A1* (Proteintech, 14327-1-AP); *CD29*-PE (eBioscience, 12-0291-81); *CD26*-PerCP-Cyanine5.5 (eBioscience, 45-0261-82).

The sequences of siRNA *SLCO1B3*siRNA: 5′- AUGCCACUGAAUUAUUUCCTT -3′ ; *SLCO2A1*siRNA: 5′-UUGCUUCAUCUGCUGUGGCTT -3′; nonspecifc siRNA: 5′-ACGUGACACGUUCGGAGAATT -3′

Supplemental Figures

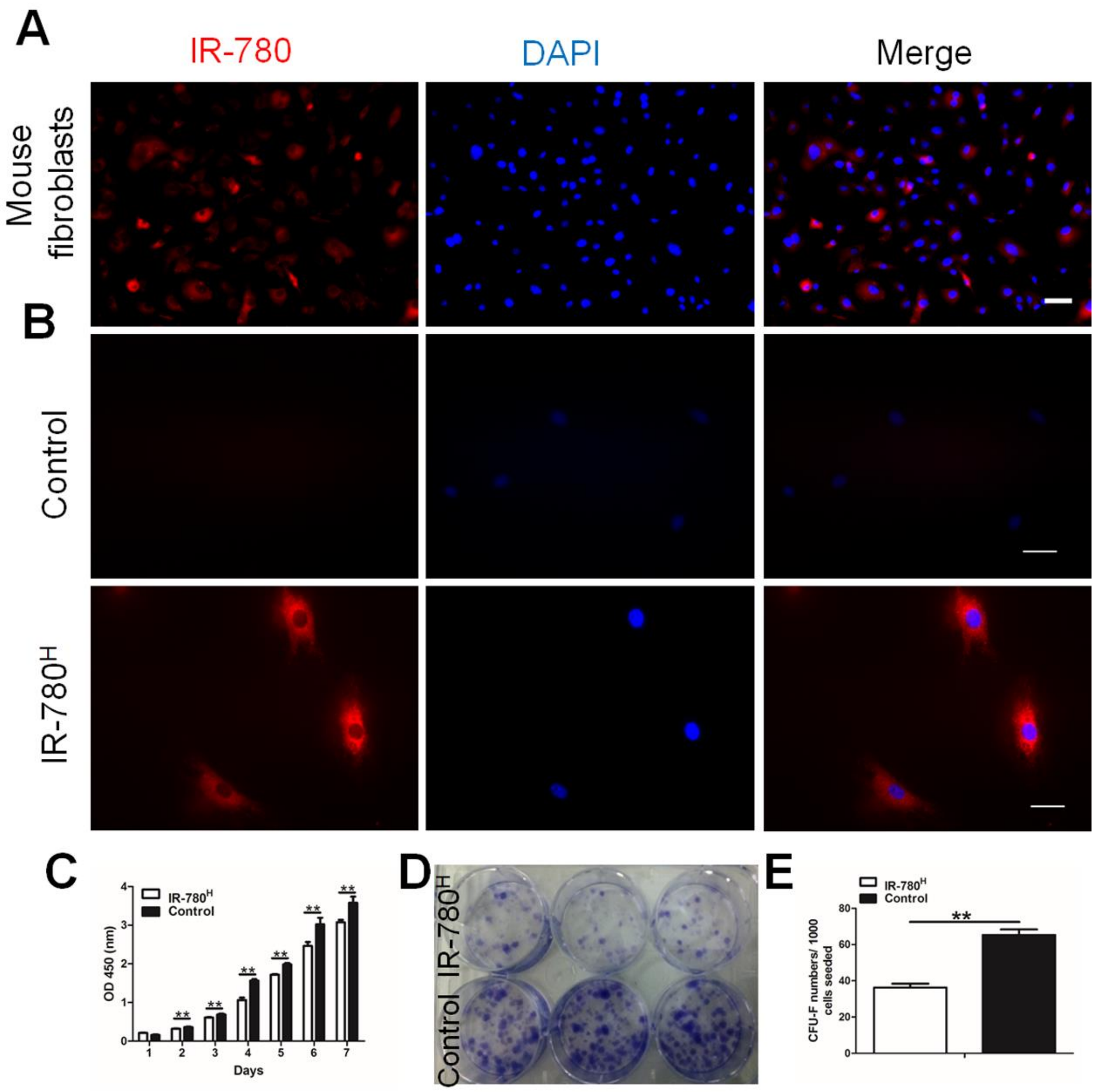

Figure S1. Characterization of IR-780^{H} fibroblasts. (A) The differentially accumulation of IR-780 in the mouse skin fibroblasts. Scale bar, 75 μm. (B) NIR images of newly isolated mouse IR-780^{H} and control fibroblasts. Scale bar, 75 μm. Analysis of (C) proliferation and (D, E) colony forming abilities of human IR-780^{H} fibroblasts compared to control fibroblasts. **, P<0.01.

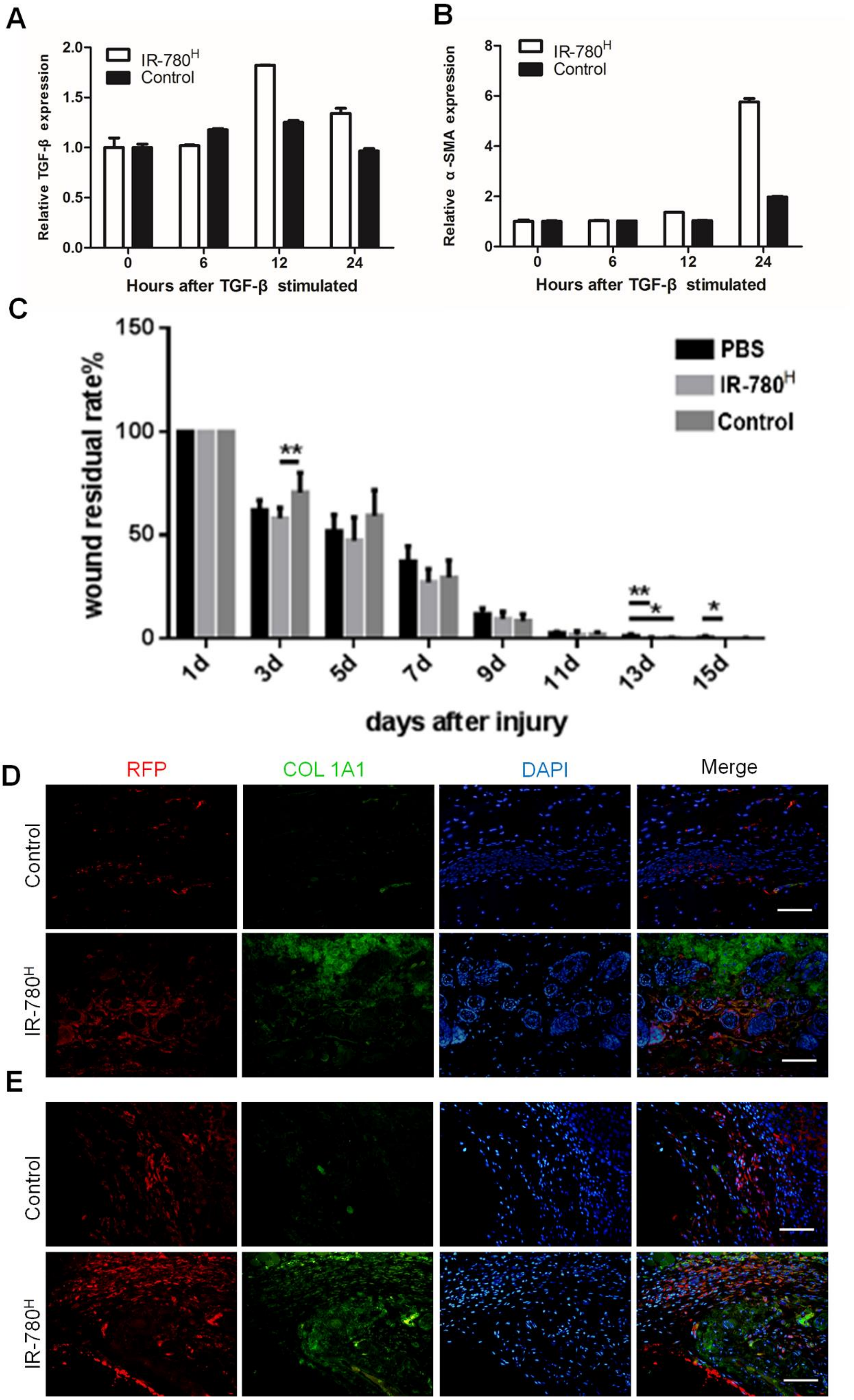
A
Relative TGF-β expression
IR-780H
Control
Hours after TGF-β stimulated
B
Relative α -SMA expression
IR-780H
Control
Hours after TGF-β stimulated
C
wound residual rate%
PBS
IR-780H
Control
days after injury
D
RFP
COL 1A1
DAPI
Merge
Control
IR-780H
E
Control
IR-780H

Figure S2. Transplantation of IR-780^{H} fibroblasts into different fibrosis models. Expression of (A) TGF-β1 and (B) α-SMA in IR-780^{H} human fibroblasts with TGF-β1 stimulation. (C) wound healing rate in wounded rats transplanted with IR-780^{H} and control human fibroblasts. *, $P<0.05$.**, $P<0.01$. Immunostaining of RFP and COL1A1 in (D) mouse skin tissues receiving subcutaneous transplantation of IR-780^{H} and control neonatal ROSA26mTmG fibroblasts and (E) mouse tumor tissues receiving subcutaneous transplantation of IR-780^{H} or control neonatal ROSA26mTmG fibroblasts with B16-F10 mouse melanoma cells. Scale bar, 100 μm.

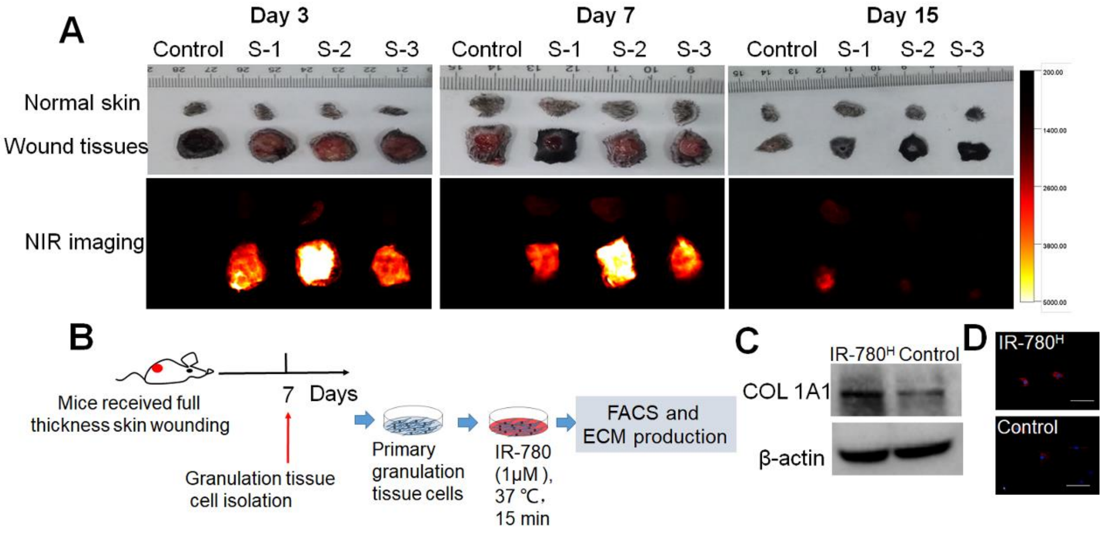

Figure S3. Localization of IR-780 in the wound tissues and isolation and characterization of IR-780^{H} granulation tissue cells. (A) NIR images of wound tissues at 3,7,15 days following wounding with pre-treated by IR-780. (B) Schematic of the experimental strategy for isolation and characterization of IR-780^{H} granulation tissue cells. (C) Western blot analysis and (D) Immunostaining of IR-780^{H} granulation tissue cells for COL1A1. Scale bar, 100 μm.

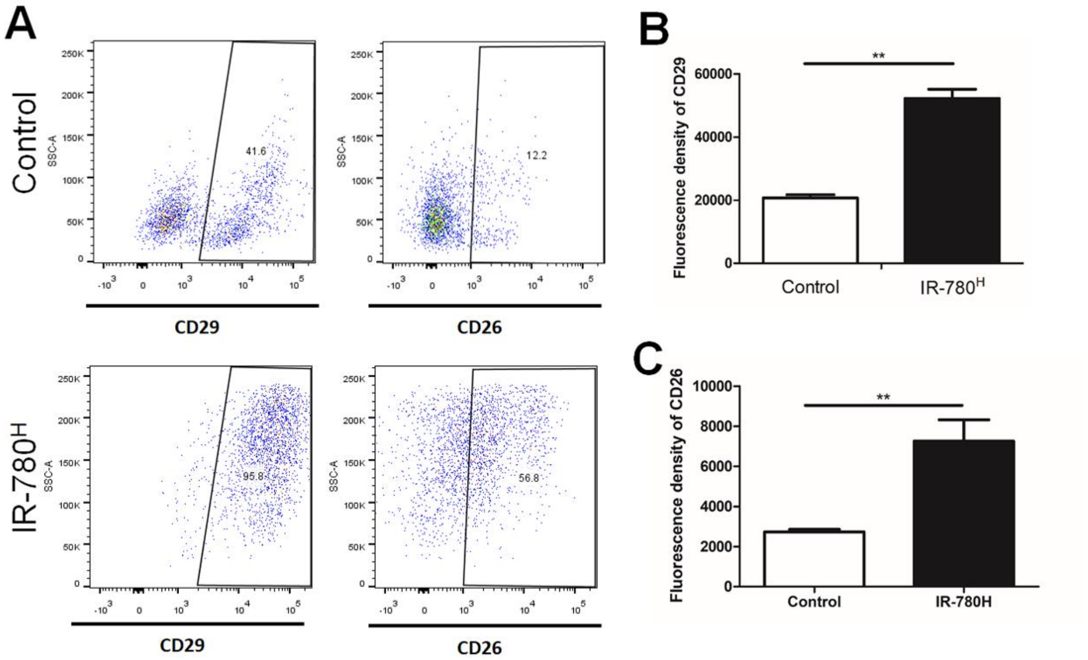

Figure S4. Expression of surface markers in IR-780^H granulation tissue cells. (A, C) The expression of CD29, CD26 in IR-780^H granulation tissue cells and control granulation tissue cells at 7 days following wounding.(B,C) the expression levels of surface marker CD29 and CD26 in IR-780^H granulation tissue cells and control granulation tissue cells at 7 days following wounding.**, P<0.01.

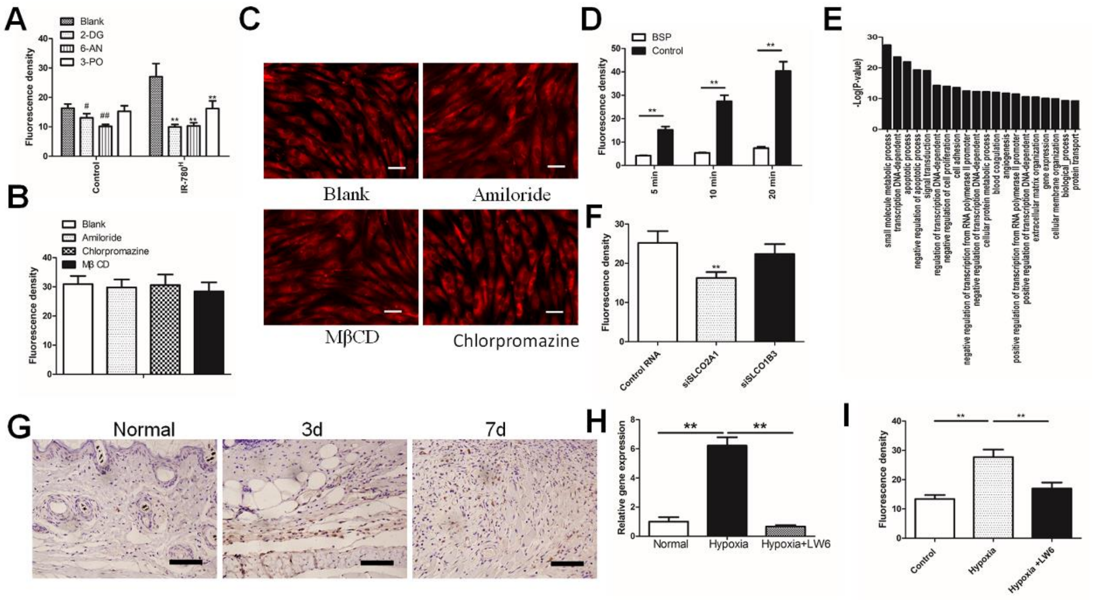

Figure S5. The uptake of IR-780 in fibroblasts is dependent on SLCO2A1 regulated by HIF-1 α(A) The fluorescent intensity in human IR-780^H fibroblasts and control fibroblasts treated with glycolytic inhibitors (2-DG,6-AN,3PO).(B,C) The fluorescent intensity in fibroblasts treated with endocytotic inhibitors(chlorpromazine, amiloride, MβCD). (D) The fluorescent intensity in human IR-780^H fibroblasts and control fibroblasts treated with BSP.(E) The upregulated biological processes in human IR-780^H fibroblasts compared to control fibroblasts. (F) The fluorescent intensity in fibroblasts treated with siRNA of SLCO2A1 and SLCO1B3. (G)The expression of HIF-1α in wounded tissues at 3, 7 days following wounding. (H) The gene expression of SLCO2A1 in fibroblasts cultured in hypoxia (5%O_2) with or without LW6. (I) The fluorescent intensity in fibroblasts cultured in hypoxia (5%O_2) with or without LW6. Scar bar, 100 µm. **, P<0.01.

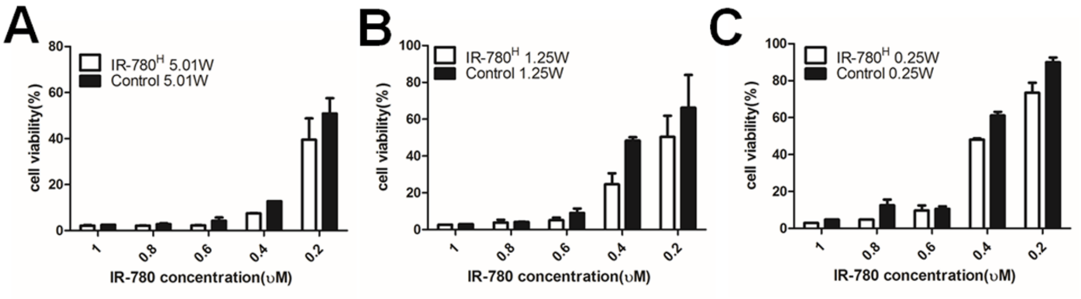

Figure S6. The photothermal, photodynamic effects of IR-780 in vitro (AtoC) Cell viability testing of human IR-780^{H} and control fibroblasts exposed to different IR-780 concentration and irradiation power.

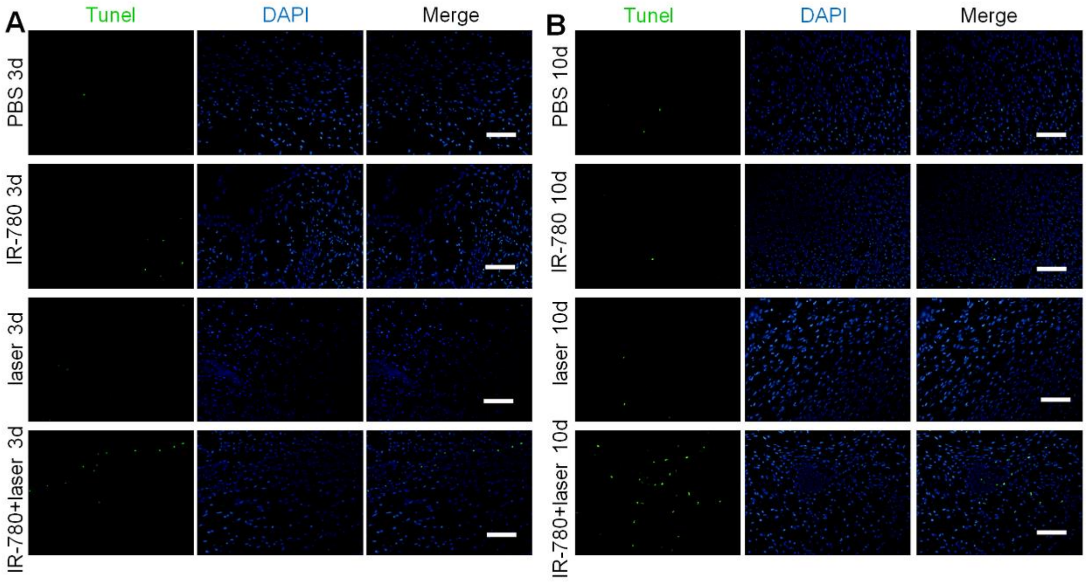

Figure S7. Low dose of IR-780 irradiated with NIR laser induced enhanced cell death in wound tissues. TUNEL staining of wound tissues treated with PBS, IR-780, laser or IR-780+laser at (A) 3, (B)10 days following wounding. IR-780 were administrated as described in 'Figure6C') Scale bar, 100 µm.

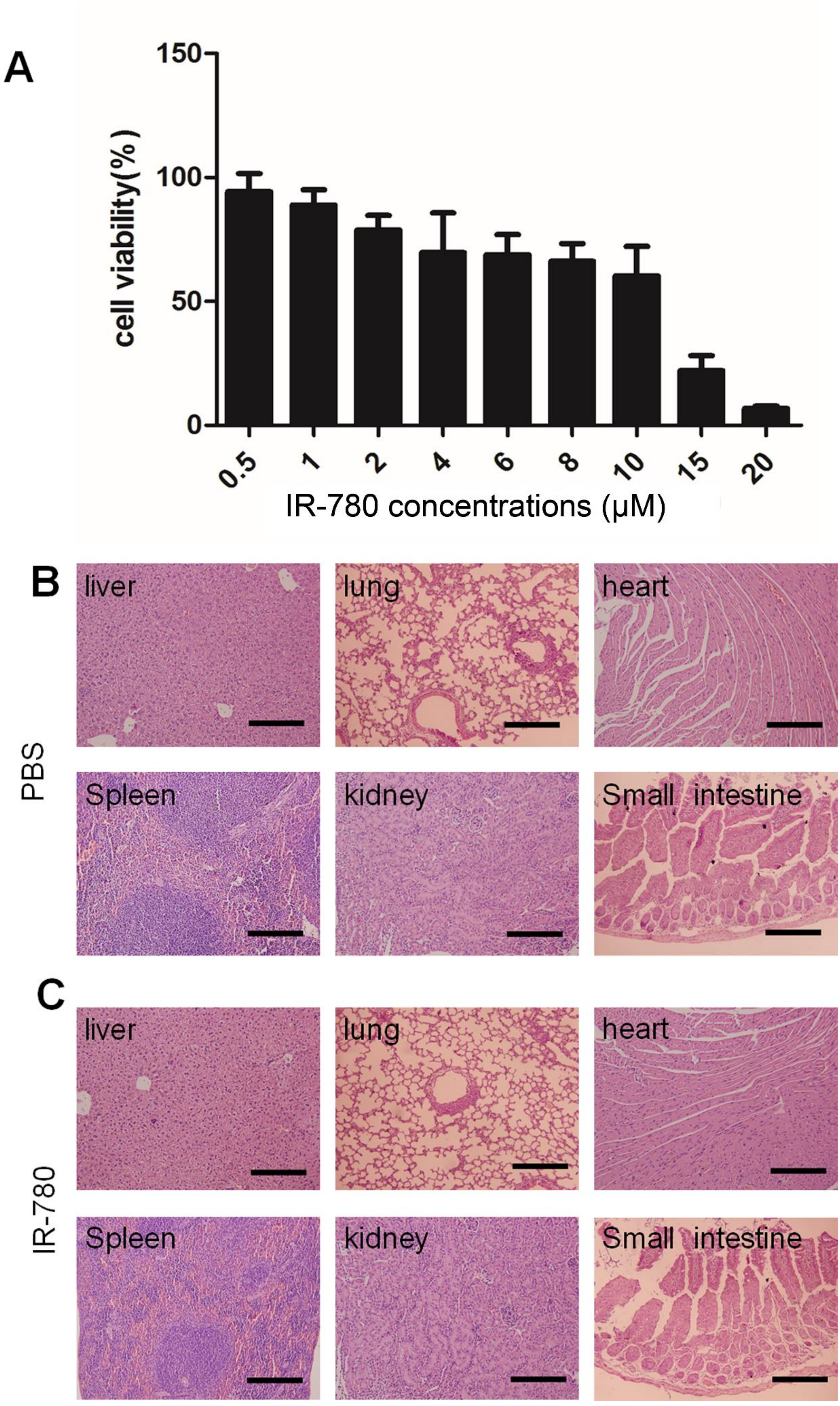
A
150
100
50
0
cell viability(%)
0.5
1
2
4
6
8
10
15
20
IR-780 concentrations (μM)
B
PBS
liver
lung
heart
Spleen
kidney
Small intestine
C
IR-780
liver
lung
heart
Spleen
kidney
Small intestine

Figure S8. Toxicity evaluation of IR-780 (A) cell viability testing of human fibroblasts exposed to different IR-780 concentrations. HE staining of organs in mice treated with (B) PBS and (C) IR-780 (1.334mg/kg, thrice injection as in ‘Figure6C’). Scale bar, 100 μm.
